# Quantum Transport in a Silicon Nanowire FET Transistor: Hot Electrons and Local Power Dissipation

**DOI:** 10.3390/ma13153326

**Published:** 2020-07-26

**Authors:** Antonio Martinez, John R. Barker

**Affiliations:** 1 College of Engineering, Swansea University, Engineering East, Fabian Way, Crymlyn Burroughs, Swansea SA1 8EN, UK; 2 James Watt School of Engineering, College of Science and Engineering, University of Glasgow, Glasgow G12 8LT, Scotland, UK; john.barker@glasgow.ac.uk

**Keywords:** silicon nanowires, nano-transistors, quantum transport, hot electrons, self-cooling, nano-cooling, thermoelectricity, heat equation, non-equilibrium Green functions, power dissipation

## Abstract

A review and perspective is presented of the classical, semi-classical and fully quantum routes to the simulation of electro-thermal phenomena in ultra-scaled silicon nanowire field-effect transistors. It is shown that the physics of ultra-scaled devices requires at least a coupled electron quantum transport semi-classical heat equation model outlined here. The importance of the local density of states (LDOS) is discussed from classical to fully quantum versions. It is shown that the minimal quantum approach requires self-consistency with the Poisson equation and that the electronic LDOS must be determined within at least the self-consistent Born approximation. To bring in this description and to provide the energy resolved local carrier distributions it is necessary to adopt the non-equilibrium Green function (NEGF) formalism, briefly surveyed here. The NEGF approach describes quantum coherent and dissipative transport, Pauli exclusion and non-equilibrium conditions inside the device. There are two extremes of NEGF used in the community. The most fundamental is based on coupled equations for the Green functions electrons and phonons that are computed at the atomically resolved level within the nanowire channel and into the surrounding device structure using a tight binding Hamiltonian. It has the advantage of treating both the non-equilibrium heat flow within the electron and phonon systems even when the phonon energy distributions are not described by a temperature model. The disadvantage is the grand challenge level of computational complexity. The second approach, that we focus on here, is more useful for fast multiple simulations of devices important for TCAD (Technology Computer Aided Design). It retains the fundamental quantum transport model for the electrons but subsumes the description of the energy distribution of the local phonon sub-system statistics into a semi-classical Fourier heat equation that is sourced by the local heat dissipation from the electron system. It is shown that this self-consistent approach retains the salient features of the full-scale approach. For focus, we outline our electro-thermal simulations for a typical narrow Si nanowire gate all-around field-effect transistor. The self-consistent Born approximation is used to describe electron-phonon scattering as the source of heat dissipation to the lattice. We calculated the effect of the device self-heating on the current voltage characteristics. Our fast and simpler methodology closely reproduces the results of a more fundamental compute-intensive calculations in which the phonon system is treated on the same footing as the electron system. We computed the local power dissipation and “local lattice temperature” profiles. We compared the self-heating using hot electron heating and the Joule heating, i.e., assuming the electron system was in local equilibrium with the potential. Our simulations show that at low bias the source region of the device has a tendency to cool down for the case of the hot electron heating but not for the case of Joule heating. Our methodology opens the possibility of studying thermoelectricity at nano-scales in an accurate and computationally efficient way. At nano-scales, coherence and hot electrons play a major role. It was found that the overall behaviour of the electron system is dominated by the local density of states and the scattering rate. Electrons leaving the simulated drain region were found to be far from equilibrium.

## 1. Introduction

### 1.1. Context

**Figure 1:**
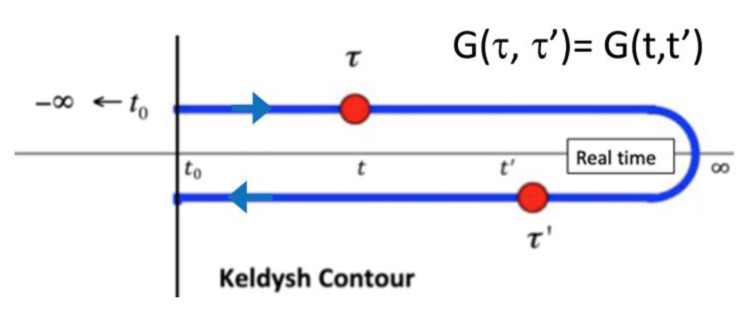
The Keldysh Contour. The general single particle Green function is the expectation value G(r,r’,τ,τ’) ≡ −*i*<T_Keldysh_*ψ*(r,*τ*)*ψ*^†^(r’,*τ*’)>_0*eq*_ where the time-ordering operator orders the field operators according to the temporal positions *τ*, *τ’* on the Keldysh contour. The temporal values of *τ*, *τ’* are denoted by the corresponding real times t, t’. In the figure are shown the temporal positions and values for the “lesser” Green Function defined for all times *τ* on the upper branch (C^−^), and for all times τ′ on the lower branch (C^+^), G^<^(r,r’,τ,τ’) ≡ −*i*<T_Keldysh_*ψ*(r,*τ*)*ψ*^†^(r’,*τ*’)>_0*eq*_.

Silicon metal-oxide semiconductor-field effect transistors (MOSFETs) have underpinned the last fifty years of the microelectronics revolution. The scalability of the basic planar single gate MOSFET has already approached the long-anticipated physical limits to device performance that require considerable architectural ingenuity to overcome. These limits include short-channel effects (SCE) such as: quantum mechanical tunnelling between source and drain and between drain and substrate; drain-induced barrier lowering (DIBL); threshold voltage roll-off. However, since the supply voltage has not been downscaled as smaller feature sizes have been achieved, the power densities have increased close to the limit of 150 W·cm^−2^ beyond which air cannot cool the device temperature [1]. The move to a 3D FinFET architecture has given some respite [2,3]. Electronic devices at nanometre scales have already been fabricated and tested [4,5,6,7]: effective gate lengths of some of the recently produced transistors are well under 20 nm [8]. There is a consensus emerging that the most promising successor to the planar MOSFET is the gate-all-around nanowire-FET (NWFET) (a cross-sectional model is displayed in Figure 1 of Section 3); it has far superior electrostatic control to the FinFET [9]. Silicon NWFETs have been fabricated with circular cross sections with diameters in a range of 9 to 1 nm [7]. These devices show very good performances and ideal sub-threshold slopes (60 mv/dec). They also showed a substantial decrease in the on-current as the cross section decreases that cannot be explained by the reduction in area. Instead, it was suggested that increases in scattering rates or the enhancement of other scattering mechanism such as surface scattering or stresses in the fabrication might be responsible. In a recent study [10], we have shown that NWFETs with ultra-scaled square cross section and circular cross-section have very similar properties.

### 1.2. Electro Thermal Properties of NWFET

The gate-all-around NWFET has excellent SCE reduction at very small gate lengths (~10 nm) but requires small diameters (~5 nm) for best performance. At these scales, however, there are issues with atomistic effects (the effect of the random discrete nature of dopants and defects [11]) but the major future issue has been long-known: any conventional electronic logic is ultimately limited by heat dissipation [12]. Comparatively few studies have been made of the electro-thermal properties of NWFETs. It is known that silicon nanowire structures have a reduced thermal conductivity compared to bulk [13,14] due to boundary scattering of phonons at the oxide sheath-silicon channel interface, which further exacerbates self-heating and hence device performance. Such devices are expected to be prone to the appearance of self-heating effects and localised hot spots. Unfortunately, the small dimensions of ultra-scaled nanowires provides a serious challenge to measurements of self-heating effects and local hot spots (although recent progress in scanning thermometry is promising [15,16]). 

### 1.3. Need for Efficient Modelling

Fabrication of nano-devices is of high lithographic precision and it is expensive. These factors make a comprehensive experimental investigation of a wide range of prototype devices prohibitive. From a design point of view, there is thus scope for good fast physics-based device modelling tools for NWFETs that assists the control of adverse electro-thermal effects. Good theoretical analysis of the electrical and carrier transport properties of proposed nanowire devices is required prior to optimised design and fabrication; in particular, the understanding of the interaction between the carrier flow and the electrostatic control. 

The simulation of the thermal performance, power dissipation and carrier lattice interaction are also important in the design of self-cooling and heat management of nanostructures. High channel temperatures lead to raised temperatures in the interconnects at the silicon source extension-metal contact [17]. The scope for conduction cooling via the source/drain/interconnects [18,19,20] has been appreciated for some time [21]. The number of research programmes on nano-cooling is rising [22]. In addition, research into the impact of quantum effects on the thermodynamic efficiency of nanostructures [22] has been a long-standing concern. 

### 1.4. Review of Modelling Approaches

Electronic transport and heat transport at nano scales have been studied by a variety of methodologies. Semi-classical techniques complemented with quantum corrections such as the Drift-Diffusion (DD) approach [23,24,25,26,27] and the Monte Carlo method [28,29,30,31,32,33,34,35,36,37] have been widely used. 

The Drift-Diffusion approach is particularly computationally efficient but assumes that the electron charge fluid is both in local equilibrium [38] and moves due to electric fields and gradients of concentration. The equation to be solved is the charge density conservation law usually called the continuity equation. Generalizations of the DD approach and energy balance in concomitance with the heat equation have been carried out, a thorough investigation is presented in [39]. An extra equation can be added to the Drift-Diffusion model to treat the local changes in momentum or velocity of the charge fluid; the model that results is termed the hydrodynamic model. However, quantum effects such as confinement and tunneling need to be artificially added. 

The Monte Carlo method [28] which is founded on a particle representation of the semi-classical Boltzmann equation is ideal to treat non-equilibrium processes semi-classically; the flows are energy-resolved but the carrier or electron transport is treated classically. It is assumed that the electron de Broglie wavelength is much smaller than the scale of variation of the electrical potential. This last assumption is violated in small nanostructures. Any quantum coherent transport is neglected in the semi-classical formalism. In addition, the scale of the thermalisation (as will be shown in this paper) is much larger that the dimension of the nanostructure. 

A powerful quantum transport methodology, the Keldysh non-equilibrium Green function (NEGF) formalism, was applied from the 70’s in order to describe non-equilibrium quantum transport process beyond the linear response in nanostructures [40]. The formalism was used to study high electric field effects [41]. The original formalism was developed in the 60’s by Schwinger [42], Keldysh [43], Kadanov and Baym [44] to address highly non-equilibrium processes such as those in Masers. The formalism uses matrix equations containing a mix of dynamical and kinetic Green functions [43].

The transport methodology satisfies the second law of thermodynamic as the entropy generated by the transfer of electrons from one reservoir to another reservoir is always positive [22]. The non-equilibrium scattering rates are self-consistently dependent on the local electric field (or equivalently the local electrostatic potential), which is not the case for Boltzmann transport. This feature was pointed out using a super-resolvent non-linear time-dependent quantum kinetic equation based on the density matrix formalism [45,46,47], and confirmed in later studies by the NEGF formalism [41]. Electro-thermal simulations have been carried out using a variety of methodologies ranging from semiclassical [48], a blend of quantum transport with classical heat equation to a pure quantum mechanical [49] and atomistic methodologies [50]. All these methodologies have their weaknesses and strengths. The semi-classical methodologies are very efficient and handle micrometer size devices however they ignore coherence, tunneling and renormalization of the density of states and therefore lose accuracy for devices of few tens of nanometers. The pure atomistic methodologies (mostly based in the NEGF formalism) consider the wave nature of electrons, coherence and quantum effects that require substantial computational power, and it is computationally feasible only for very small dimensions. However, effects and boundary conditions that are far from the active device region could affect the electrical and heat performance of the device, for example, remote optical phonon scattering and remote coulomb interactions [51,52,53,54], phonon reflections or transmission through interfaces [55,56].

### 1.5. Content of This Paper

In the present paper, Section 2 provides a brief review and perspective on the key concepts and the transition from semi-classical to fully quantum mechanical modelling of electro-thermal effects applicable to quasi-one dimensional NWFETs. Section 2.1 discusses the basic concepts of electro-thermal modelling. Section 2.2 describes the crucial local density of states in energy and the need for a transition from semi-classical to full quantum transport for electro-thermal device modelling. Section 2.3 gives a brief overview of the non-equilibrium Green function (NEGF) methodology augmented by explicit technical details in an Appendix (Appendix A discusses the local density of states for nanowires and the multi-valley picture; Appendix B describes how contact self-energies are used to project the open many-body problem into a finite device simulation domain; Appendix C discusses details of the Keldysh NEGF formalism; Appendix D gives explicit details of the phonon Green functions and the generic electron-phonon self-energies; Appendix E demonstrates explicitly that the NEGF formalism shows no heat-dissipation for elastic acoustic phonon scattering; Appendix F describes detailed balance within the NEGF formalism; Appendix G describes the importance of the Kramers-Kronig relations for self-energies). 

In Section 3, we present our simulation methodology for the silicon gate-all-around nanowire FET that uses the NEGF picture for the electrons coupled to a semi-classical Fourier heat equation. The device structure and the quasi-1D representation is introduced in Section 3.1. The NEGF methodology for the density and current density is outlined in Section 3.2. Section 3.3 discusses energy balance and power. Section 3.4 presents the results and discussion including comparison with other simulated cases. Section 3.5 discusses perspectives and challenges. Finally, the conclusions are presented in Section 4. In the following we exclude radiative emission and absorption as this is not relevant for conventional integrated circuits, although this is relevant to photo-excited hot electron phenomena. 

## 2. Methodology and Models

### 2.1. Basic Concepts of Electro-Thermal Modelling

The key quantity in electro-thermal phenomena is the local energy current density *J_Energy_*(r,*t*) synonymous with the heat current density. If we consider a finite volume Ω bounded by an enclosing surface *S* then the total power generated within the volume Ω is balanced by the net integrated energy current density flowing out of the surface *S:*

(1) $${∭}_{\Omega }\;P\left(r,t\right)d^{3}r=-\iint_{S}J_{Energy}\left(r,t\right)\cdot dS$$

where P(r,t) is the local power density, synonymous with the local rate of heat generation. Applying the divergence theorem we obtain:

(2) $$P\left(r,t\right)=-\nabla \cdot J_{Energy}\left(r,t\right)$$

If Ω represents the active volume of an FET device there is no net heat generation in the device when it is switched off as it is in thermal equilibrium with the lattice and its environment. If the device is switched on (gate voltage applied) and a (chemical potential difference) voltage difference established between the source and drain contacts then an electrical current flows characterised by the current density *J*(r,*t*) = *ej*(r,*t*) where *j*(r,*t*) is the particle current density.

In a purely ballistic channel [57,58] for which the electron flow is quantum coherent (no dissipative scattering), the electron transport involves a net injection of electrons at high energy and momentum from the source followed by net extraction at the drain where the excess energy and momentum are dissipated: the flow is quantum transmission and the conductance *G* = 1/*R* (*R*:The electrical resistance) of the channel is quantised in units of *e*^2^/*πħ*. This extreme case (best studied for low temperatures) illustrates that quantum transport can lead to hot spots, in this case strong heating occurs in the drain contact. If a defect or impurity occurs in a ballistic channel such that its interaction with an electron is representable as a simple scalar potential perturbation, the flow then involves reflection and tunnelling transmission that may be described by *T_c_* the transmission coefficient for the channel. Then *G_min_* = *T_c_e*^2^/*πħ* which is Landauer’s formula [59]. If *T_c_* < 1 there is elastic (potential) scattering in the channel. Again, the dissipation of energy is in the contact region; the dissipation of momentum is partly within the channel via the defect/impurity. This simple picture ignores the electrical field profile E(r,*t*) = −▽*ϕ*(r,*t*) in the channel. In practice, it is neither abrupt nor uniform; instead it may be determined self-consistently from Poisson’s Equation:

(3) $$\nabla \cdot \varepsilon_{d}\nabla \phi \left(r,t\right)=-\rho (r,t)$$

where *ρ* is the charge density in the channel and *ε_d_* is the dielectric permittivity.

Silicon nanowire transistors do not operate in the purely ballistic regime although elements of the simple transmission/reflection flows may occur as we discuss later. In general, the applied electric field supplies energy and momentum to the carriers throughout the device and the electrostatic potential varies smoothly between source and drain. For dissipative transport the energy and momentum gained locally from the field will be transferred to the phonon system by scattering within the device involving a loss-gain process in which phonons may be emitted or absorbed by the electron assembly. Inelastic scattering gives rise to energy dissipation whereas the momentum dissipation occurs for elastic and inelastic processes. 

More detailed non-phenomenological analysis requires an electron transport formalism and ultimately a coupled phonon transport picture. The deepest semi-classical picture is provided by the well-known Boltzmann transport equation for the electron probability distribution *f*[r,k,*t*]:

(4) $$\frac{\partial f\left[r,k,t\right]}{\partial t}+v\left(k\right)\cdot \frac{\partial f\left[r,k,t\right]}{\partial r}+eE\left(r,t\right)\cdot \frac{\partial f\left[r,k,t\right]}{\partial \hbar k}=-\frac{\partial f\left[r,k,t\right]}{\partial t}{|}_{s}$$

Here the velocity v is determined from the k-dependence of the conduction band energy *ε*(k):

(5) $$v(k)=\frac{1}{\hbar }\frac{\partial \varepsilon \left(k\right)}{\partial k}$$

The second term in Equation (4) represents diffusive effects, the third term represents the drift of the distribution induced by an applied electric field, the RHS represents the change in *f* due to scattering processes. The scattering integral (actually a sum over k-states) is given by

(6) $$\frac{\partial f\left[r,k,t\right]}{\partial t}{|}_{s}=\underset{k{}^{\prime }}{\sum }\left\{f\left[r,k{}^{\prime },t\right]\left(1-f\left[r,k,t\right]\right)R\left[r,k{}^{\prime },k,t\right]-f\left[r,k,t\right]\left(1-f\left[r,k{}^{\prime },t\right]\right)R\left[r,k,k{}^{\prime },t\right]\right\}\;$$

We shall be mostly concerned with phonon scattering for which the scattering rate from k to k’ may be written as a sum over the phonon modes *b* as:

(7) $$\begin{array}{cc} R\left[r,k,k^{\prime },t\right]= & \sum_{b\;K}\Lambda_{b}\left[k,k^{\prime }\right]\left\{\delta_{k-q-k^{\prime },K}\left(f_{\mathrm{phonon}b}\left[r,q,t\right]+1\right)\delta \left(\varepsilon \left(k^{\prime }\right)-\varepsilon \left(k\right)+\hbar \omega_{\mathrm{bq}\;}\right)\right.\\ \; & \left.+\delta_{k+q-k^{\prime },K}f_{\mathrm{phonon}b}\left[r,q,t\right]\delta \left(\varepsilon \left(k^{\prime }\right)-\varepsilon \left(k\right)-\hbar \omega_{bq}\right)\right\} \end{array}$$

Here Λ*_b_* is the scattering kernel, *K* represents a reciprocal lattice vector for Umklapp processes, *ħω_b_*_q_ is the energy of the *b* mode phonon at wave vector q. The delta-functions in energy and momentum represent the conservation of energy and momentum within a scattering event. The function *f*_phonon b_[r,q,*t*] is the probability distribution function for a phonon in mode *b* with wave vector q. The scattering rates are local in space and time, and evaluated in the Born approximation and do not depend on the applied electrostatic field. The first term in the brackets {…} of Equation (7) represents electron scattering with the emission of a phonon; the second term describes absorption of a phonon. If the phonon distributions are in internal thermal equilibrium at a well-defined lattice temperature *T* they assume the form of a Bose-Einstein distribution:

(8) $$f_{\mathrm{phonon}\;b}\left[r,q,t\right]\to (e^{\frac{\hbar \omega_{bq}}{k_{B}T}}-1)$$

The Boltzmann transport equation is usually solved numerically by using the Monte-Carlo method [28]. Let us now specialise to the steady-state electro-thermal problem for which $\frac{\partial f}{\partial t}=0$.

The local kinetic energy current density is given by:

(9) $$J_{kinetic}\left(r\right)=\overset{\;}{\underset{k}{\sum }}\varepsilon \left(k\right)j\left(r,k\right)\to \int d^{3}k\;\rho_{k}\;\varepsilon \left(k\right)j\left(r,k\right)=\int d^{3}k\;\rho_{k}\;\varepsilon \left(k\right)v\left(k\right)f\left[r,k\right]$$

where the summation over k is replaced by integration by introducing $\rho_{k}$ the density of k-states.

We may obtain an expression for J*_kinetic_*(r) by multiplying the steady-state version of Equation (4) by *ε*(k) and summing over all k to obtain after an integration by parts and re-arranging the collision integral:

(10) $$\nabla \cdot J_{kinetic}\left(r\right)-E\left(r\right)\cdot J\left(r\right)=-\overset{\;}{\underset{k}{\sum }}\varepsilon \left(k\right)\frac{\partial f\left[r,k\right]}{\partial t}{|}_{s}$$

(11) $$\begin{array}{cc} =\sum_{k}\sum_{k^{\prime }}\left\{\varepsilon \left(k\right)-\varepsilon \left(k^{\prime }\right)\right\}f\left[r,k\right]\left(1-f\left[r,k^{\prime }\right]\right)R\left[r,k,k\right] & =-P(r) \end{array}$$

(12) $$P\left(r\right)=-\nabla \cdot J_{Energy}\left(r\right)=-\nabla \cdot J_{kinetic}\left(r\right)+E\left(r\right)\cdot J\left(r\right)$$

where from Equation (7) *ε*(k’)–*ε*(k) is the energy transferred to or absorbed from the lattice per unit time when an electron scatters from state k’ to state k by emitting or absorbing a phonon. The explicit expression for the local power density *P*(r) is

(13) $$\left.P\left(r\right)=\sum_{k}\sum_{k^{\prime }}\left\{\varepsilon \left(k^{\prime }\right)-\varepsilon \left(k\right)\right\}f\left[r,k\right]\left(1-f\left[r,k^{\prime }\right]\right)R\left[r,k,k^{\prime }\right]\right.$$

showing that for *elastic* scattering processes *ε*(k’) = *ε*(k), the contribution to the power density vanishes: no dissipation. Re-calling our discussion of ballistic transport it is evident from Equation (13) that in semi-classical transport the heat dissipation must occur where the scattering rate R[r,k,k’] is non-zero.

Equation (12) makes the Joule heating explicit, where E(r) is the local electrical field and J(r) = ej(r) is the electrical current density. The Joule power density E.J when integrated throughout the device produces the familiar Joule power I V. The first term on the RHS of Equation (12) tracks the spatial variation of the “kinetic energy” current density of the electron ensemble. Crucially, if the kinetic energy current density J*_kinetic_*(r) does not change with position r the electron energy distribution will follow the bending of the electrostatic potential and the electron mean kinetic energy will not increase relative to the conduction band edge; therefore the energy distribution of the electrons will remain in local equilibrium, there will be no hot electron phenomena. On the other hand, if there is a large change in the spatial dependence of the kinetic energy current density there will be heating or cooling of the electronic sub-system. 

Equations (9) and (10) lead directly to the thermodynamically significant detailed balance equations and have much wider generality than the classical picture.

The recognised route to simulating devices operating under non-isothermal conditions is to add a transport model to a standard heat equation. Using the local heat generation rate *P*(r,*t*) as the source of excess heat we obtain the heat equation (Fourier’s Equation) for the local lattice temperature *T*(r,*t*):

(14) $$C_{h}\frac{\partial T\left(r,t\right)}{\partial t}=\nabla \cdot \kappa \nabla T\left(r,t\right)+P\left(r,t\right)$$

where $C_{h}$ is the total heat capacity and *κ*(r,*t*) is the local thermal conductivity.

For steady-state simulations Equation (8) reduces to:

(15) ∇·κ∇T(r)=−P(r)

There are three main approaches to specify the heat source term *P*: (i) the Joule-heating model, exemplified by local equilibrium carriers commonly used in drift-diffusion modelling; (ii) assuming non-equilibrium electrons (hot electrons) interacting with equilibrium phonons; (iii) non-equilibrium electrons interacting with local equilibrium phonons. 

(i) If we neglect electron-hole recombination and generation processes the Joule heating model [60] assumes:

(16) P(r,t)=E(r,t)·J(r,t)

where E is the local electric field and J is the local electrical current density.

Evidently, the highest heating rate will occur where the scalar product is at a maximum. In conventional FETs most studies indicate that the maximum heating occurs under the gate where most of the potential drop takes place and where the electrical current density is the largest. 

(ii) Here, we require an Equations (12)–(14) which picks up the transfer of energy from the electron system to the phonons in the local lattice. If the phonons are assumed to be in internal thermal equilibrium at temperature *T* then we can evaluate the net energy loss via the RHS of Equation (14) by using the matrix-elements and phonon distribution functions within the scattering rates *R* of Equation (7) by their equilibrium values [12,61]. More generally, we may evaluate a local lattice temperature *T*(r) (from self-consistent solution of the coupled heat balance equation [62,63]). 

(iii) To obtain full generality [34,35,36,64,65,66,67,68,69], we must introduce and solve self-consistently the full set of phonon transport equations for each phonon type at each location in the system including internal equilibrium processes such as phonon-phonon scattering and external processes such as boundary scattering. If the phonon distributions are parameterised by a different non-equilibrium value *T_b_*(r) for each mode *b* then it is still relatively easy to proceed; if not, the problem becomes a major challenge.

Further progress is only possible by resorting to a full space-dependent, energy-resolved quantum statistical approach to which we now turn.

### 2.2. LDOS and the Transition from Classical to Quantum Modelling

Consideration of the local energy density of states (LDOS) provides a clear example of the need for a transition to a quantum description of electro-thermal transport. Semi-classically, one assigns the particle momentum p to a wave vector k by p = *ħ*k, where the underling implication of a plane wave state exp[ik·r] only has meaning in the evaluation of the scattering rate matrix elements in the collision integral. Its amplitude is normalised to unity. The local density of states is then defined as:

(17) $$\rho_{LDOS}\left(r,\varepsilon \right)=\underset{k}{\sum }\;\delta \left(\varepsilon -\varepsilon \left(k\right)-V\left(r\right)\right)=\int d^{3}k\;\rho_{k}\delta \left(\varepsilon -\varepsilon \left(k\right)-V\left(r\right)\right)$$

**Figure A1:**
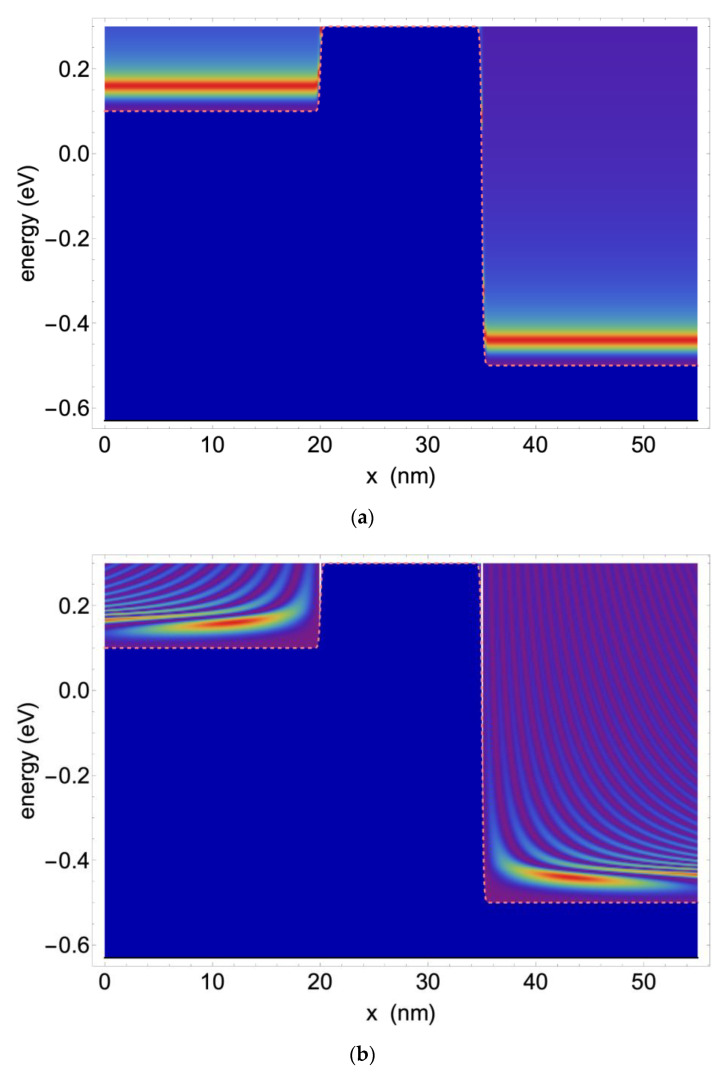
LDOS for a silicon nanowire-FET (NWFET) simple device potential profile (dashed pink line) (**a**) classical result; (**b**) quantum result showing influence of strong reflections on either side of the barrier.

Noting that *ρ_k_* = 2*L^N^*/(2π)*^N^* where *N* is the number of dimensions and *L* is the normalisation length, Equation (14) is easily evaluated for 3D, 2D and 1D systems as:

(18) $$\rho_{LDOS}^{3D}\left(x,y,z,\varepsilon \right)=\left(\frac{m}{\pi^{2}\hbar^{2}}\right)\sqrt{\frac{2m\left(\varepsilon -V\left(x,y,z\right)-\varepsilon_{c}\right)}{\hbar^{2}}}$$

(19) $$\rho_{LDOS}^{2D}\left(x,y,\varepsilon \right)=\left(\frac{m}{\pi^{\;}\hbar^{2}}\right)\theta \left(\varepsilon -V\left(x,y\right)-\varepsilon_{c}\right)$$

(20) $$\rho_{LDOS}^{1D}\left(x,\varepsilon \right)=\left(\frac{2m}{\pi^{\;}\hbar^{2}}\right)/\sqrt{\frac{2m\left(\varepsilon -V\left(x\right)-\varepsilon_{c}\right)}{\hbar^{2}}}\;$$

where *m* is the electron mass for a simple isotropic band structure, *ε_c_* is the conduction band edge; it is assumed that the dimensionality factor *L* = 1 and θ is the unit step function. In the Appendix A, Figure A1, Equation (20) is illustrated classically and for a simple quantum generalisation for a typical band edge profile for a 1D NWFET.

The quantum origins of Equation (17) are very simple; consider for example, a general quantum Hamiltonian *H* that has a complete set of eigenvalues and eigenstates {ε*_α_*,|*α* >} with completeness condition:

(21) $$\underset{\alpha }{\sum }\left|\alpha ><\alpha \right|=I$$

where I is a unit operator. The density of states in energy per unit volume follows as

(22) $$\rho_{DOS}\left(\varepsilon \right)=\underset{\alpha }{\sum }\delta \left(\varepsilon -\varepsilon_{\alpha }\right)=\underset{\alpha }{\sum }<\alpha \left|\delta \left(\varepsilon -H\right)\right|\alpha >$$

(23) $$\rho_{DOS}\left(\varepsilon \right)=Tr\left[\delta \left(\varepsilon -H\right)\right]$$

(24) $$\rho_{DOS}\left(\varepsilon \right)=\underset{\beta }{\sum }\;<\;\beta \left|\delta \left(\varepsilon -H\right)\right|\beta >=\underset{\beta ,\alpha }{\sum }\left|<\beta \right|\alpha >{|}^{2}\;\delta \left(\varepsilon -\varepsilon_{\alpha }\right)$$

where Equation (23) shows the representation independence of the trace operation. Equation (24) re-expresses the result in terms of any basis {*ε_β_*,|*β* >} in particular if we choose the position representation {|r >}, Equation (24) leads to the local density of states

(25) $$\rho_{LDOS}\left(r,\varepsilon \right)=\underset{\alpha }{\sum }\left|<r\right|\alpha >{|}^{2}\;\delta \left(\varepsilon -\varepsilon_{\alpha }\right)=<r\left|\delta \left(\varepsilon -H\right)\right|r>$$

For a simple free electron model the eigenstates α are just k-states : <r|k> = exp[ik·r] with eigenvalues *ε*(k) and we recover Equation (14) for *V* = 0.

By using the operator identity

(26) $$\delta \left(\varepsilon -H\right)=\underset{\eta \to 0^{+}}{\lim}\;\frac{1}{2\pi i}\{{\left(\varepsilon -i\eta -H\right)}^{-1}-{\left(\varepsilon +i\eta -H\right)}^{-1}\}$$

Equation (25) may be formulated in terms of the advanced and retarded Green’s operators for the steady-state operator equation

(27) $$\left(\varepsilon +i\eta -H\right)G^{R}=I;\;\left(\varepsilon -i\eta -H\right)G^{A}=I$$

(28) ρLDOS(r,ε)=<r|δ(ε−H)r>=−12πiGR(r,r′,ε)−GA(r,r′,ε)r′→r≡A(r,ε)=−1πImGRr,r′,ε∞

In the position representation Equations (27) are matrix equations for the Green functions *G^R^*(r,r’,*ε*), *G^A^*(r,r’,*ε*); they determine the description of the energy structure of the system. 

In thermal equilibrium, the energy distribution of electronic states will follow a Fermi-Dirac distribution

(29) $$f_{0}\left(\varepsilon -\mu \right)={\left\{1+\exp\left[\frac{\left(\varepsilon -\mu \right)}{k_{B}T}\right]\right\}}^{-1}$$

so that the equilibrium particle density will be

(30) $$n_{0}\left(r,\varepsilon \right)=\underset{\alpha }{\sum }\left|<r\right|\alpha >{|}^{2}\;\delta \left(\varepsilon -\varepsilon_{\alpha }\right)f_{0}\left(\varepsilon -\varepsilon_{\alpha }-\mu \right)\;=<r|\delta \left(\varepsilon -H\right)f_{0}(\varepsilon -H-\mu N)|r>\equiv \frac{-i}{2\pi }{G_{0}^{<}\left(r,r^{\prime },\varepsilon \right)|}_{r^{\prime }\to r}$$

Here we introduce the statistical (superscript “<” denotes “lesser than”) equilibrium Green function. This is easy to evaluate if the eigenstates of *H* are known. Equations (21)–(30) provide a simple account of how the NEGF formalism arises.

However, if *H* is split into an interaction free Hamiltonian *H*_0_ (which may include the Hamiltonian for non-interacting phonons) and an interaction term *H*_int_ describing the electron-phonon interactions, then the trace operation in Equations (28) and (30) must be widened to include a trace over the phonon states to maintain completeness. A self-consistent perturbation theory therefore requires a simultaneous treatment of the states within thermal equilibrium and the dynamical evolution of the states in non-equilibrium. 

The route to doing this was laid out in [41,42,43,44] in the 1960s, but was difficult to apply, partly because of computational resources. Important technical issues and improvements were made much later [40,70,71,72,73,74]

### 2.3. The NEGF Formalism

The NEGF formalism is ultimately based on statistical quantum field theory; in the following we will draw from references [41,42,43,44,70,71,72,73,74], but we will only review the parts of that theory necessary to consider electro-thermal transport and we avoid the very technical derivations and issues. An excellent practical introduction to the NEGF as applied to devices will be found in works and references therein by Datta [57,58], background many-body perturbation theory is discussed in detail in reference [71]. The Keldysh approach is outlined in 2.3.1 but may skipped to proceed directly to the required steady-state NEGF equations that are described in Section 2.3.2. More technical details will be found in the Appendix, Sections Appendix B, Appendix C, Appendix D, Appendix E, Appendix F and Appendix G.

#### 2.3.1. The Keldysh Picture

Let us start with a generic second-quantised many-body Hamiltonian *H* = *H*_0_ + *H_int_* describing a system of interacting (via *H_int_*) electrons and phonons. Any property of the system may be represented by an operator Ξ and its statistical expectation value. < Ξ> may be computed in thermal equilibrium from the grand canonical ensemble $\rho_{eq}$ of statistical mechanics giving:

(31) $$<\;\Xi \left(t\right){>}_{eq}=\frac{Tr\left[\Xi \rho_{eq}\right]}{Tr\left[\rho_{eq}\right]};\;\rho_{eq}=\exp\left[-\frac{H-\mu N}{k_{B}T}\right]\rho_{eq}=\exp\left[-\frac{H-\mu N}{k_{B}T}\right]$$

where *T* is the absolute temperature and *µ* is the chemical potential. Here *N* represent the second-quantised number operator for excitations (electrons and phonons). The trace operation Tr[…] means sum the diagonal matrix elements of the operand in any complete set of many-body states. 

An important example of interest is the single electron equilibrium Green function that has a two-point space-time structure:

(32) $$<\Xi \left(r,r^{\prime },t,t^{\prime }\right){>}_{eq}\equiv G_{eq}\left(r,r^{\prime },t,t^{\prime }\right)\equiv <T\psi \left(r,t)\psi^{†}(r^{\prime },t^{\prime }\right){>}_{eq}$$

Equation (32) represents the probability amplitude for the insertion of an electron at (*r’*,*t’*) and the removal of an electron at (r,*t*). The operators *ψ*(r,*t*), *ψ*^†^(*r*,*t*) are anti-commuting electron annihilation and creation field operators in the Heisenberg picture (state vectors constant, operators time-dependent.) T is the Dyson time-ordering operator which for fermions re-orders the field operators so that the term with earliest time is on the right and also multiplies the result by +1 or −1 depending on whether the new order is an even or odd permutation of the original product.

Different time-orderings of Equation (32) give Green functions with different behaviour. The Green functions $G_{eq}^{<},\;G_{eq}^{>}$ (cf. Section 2.2) are the specific time-orderings:

(33) $$G_{eq}^{<}\left(r,r^{\prime },t,t^{\prime }\right)=G_{eq}^{\;}\left(r,r^{\prime },t,t^{\prime }\right)\;(t<t^{\prime })$$

(34) $$G_{eq}^{>}\left(r,r^{\prime },t,t^{\prime }\right)=G_{eq}^{\;}\left(r,r^{\prime },t,t^{\prime }\right)(t>t^{\prime })$$

(35) $$G_{eq}^{R}\left(r,r^{\prime },t,t^{\prime }\right)=-i\theta \left(t-t^{\prime }\right)<\{\psi^{†}\left(r{}^{\prime },t\prime ),\;\psi^{\;}(r^{\;},t^{\;}\right)\}{>}_{eq}$$

(36) $$G_{eq}^{A}\left(r,r^{\prime },t,t^{\prime }\right)=i\theta \left(t\prime -t\right)<\{\psi^{\;}\left(r,t),\;\psi^{†}(r^{\prime \;},t^{\prime \;}\right)\}{>}_{eq}$$

The “lesser/greater” Green functions *G^<^/G^>^* are so named by the conditions *t* < *t*’ or *t* > *t*’). Here, the brackets { , } represent the anti-commutator. 

Using the above Equations (31) and (32) it is relatively straightforward to derive Equations (28) and (30). Equation (31) plus considerable manipulation yields an important boundary condition that relates *G*^<^ to *G*^>^ in thermal equilibrium:

(37) $$G_{eq}^{<}\left(r,r^{\prime },t,t^{\prime }\right){|}_{t=0}=-\exp\left[\beta \mu \right]G_{eq}^{>}\left(r,r^{\prime },t,t^{\prime }\right)\;{|}_{t=-i\hbar \beta }\;\;\;$$

The latter is the basis for developing finite temperature thermal equilibrium perturbation theory in complex time, see reference [71]

The non-equilibrium problem is to start at some initial time t_0_ with a system (such as a device) in thermal equilibrium and then initiate an external perturbation *H*_ext_ (applied fields or temperature gradients) that drives the system into non-equilibrium. To establish an infinite order perturbation theory for interacting systems we write the full Hamiltonian as

(38) $$H=H_{0}+H_{ext}\left(t\right)+H_{int}$$

where *H*_0_ describes free excitations (electrons, phonons in our case); *H*_ext_ (*t*) describes coupling to external fields switched on *at some time* > t_0_; and *H*_int_ represents interaction between excitations (electron-phonon coupling in our case). The system density matrix satisfies the equation of motion:

(39) $$i\hbar \frac{\partial \rho }{\partial t}=\left[H,\rho \right]$$

Assuming adiabatic switching-on of the interactions, the density matrix at time *t* is in the interaction picture given by the *S*-matrix ( evolution operator):

(40) $$\rho \left(t\right)=S\left(t,-\infty \right)\rho \left(-\infty \right)S^{†}\left(t,-\infty \right)$$

(41) $$S\left(t,t^{\prime }\right)=Texp\left[-i/\hbar \int_{t^{\prime }}^{t}dt{}^{\prime \prime }\;H_{int}\left(t{}^{\prime \prime }\right)\right]$$

(42) $$S^{†}\left(t,t^{\prime }\right)=Texp\left[-i/\hbar \int_{t^{\;}}^{t\prime }dt{}^{\prime \prime }\;H_{int}\left(t{}^{\prime \prime }\right)\right]$$

The time ordering is along the real-time integration paths.

(43) $$<\Xi \left(t\right)>=Tr\left[\Xi \left(t\right)\rho \left(t\right)\right]=Tr\left[S^{†}\left(t,-\infty \right)\Xi \left(t\right)S\left(t,\infty \right)\rho \left(-\infty \right)\right]$$

In a zero-temperature many-body systems with a stable ground state |0> (vacuum state) it is possible to reduce Equation (32) to:

(44) $$<\Xi \left(t\right)>=\frac{<0\left|TS\left(-\infty ,\infty \right)\Xi \left(t\right)\right|0>}{<0\left|S\left(-\infty ,\infty \right)\right|0>}$$

where the time-integrals are along the real time axis from −∞ to ∞. The familiar many-body perturbation theory then follows using Wick’s theorem [71] that provides cancellation of divergent terms in the perturbation expansion of the numerator of Equation (33). This simplification does not hold for non-equilibrium systems. Instead, in the Keldysh formalism Equation (33) is written as

(45) $$<\Xi \left(t\right){>}_{0eq}=Tr\left[T_{\mathrm{contour}}S_{\mathrm{contour}}\Xi \left(t\right)\rho_{0eq}\right]$$

where the *S*-matrix, S_contour_, is evaluated along a time contour C _Keldysh_ that runs from −∞ to *t* (*C*^+^) and then back from t to −∞ (C^−^).

Figure 1 displays the Keldysh contour showing the arguments (red) of the contour ordered Green function G^<^(*t*,*t*’). T_contour_ is the time ordering operator on the contour C_contour_ = C_Keldysh_. By inserting the identity operator *S*(*t*,∞)S(∞,*t*) the Keldysh contour is extended from (−∞,∞) and back again: (∞,−∞).

In Equation (45) *ρ*_0*eq*_ is the equilibrium density matrix for non-interacting excitations. The subsequent time-dependent many-body infinite perturbation theory then follows a similar Feynman diagram expansion as for zero temperature theory but involves integrations along the Keldysh contour. The perturbation theory is most easily derived using equations of motion for a hierarchy of Green functions.

In the steady-state, neglecting initial state correlations and transients, and choosing a time-independent external perturbation *H_ext_*, the basic single-particle electron Green functions may be expressed in the Heisenberg picture:

(46) $$\Xi \left(t\right)=\exp\left[\frac{i\left(H_{0}+H_{ext}+H_{int}\right)\left(t-t_{0}\right)}{\hbar }\right]\Xi \left(t_{0}\right)\exp[\frac{-i\left(H_{0}+H_{ext}+H_{int}\right)\left(t-t_{0}\right)}{\hbar }$$

where now the Hamiltonian *H* has been augmented to include the perturbing interaction *H_ext_*.

In the case of the single particle electron Green function *G*(r,r’,*t,t*’) we find:

(47) $$G_{\;}\left(r,r^{\prime },t,t^{\prime }\right)\equiv -i<T_{\mathrm{Keldysh}}\psi \left(r,t)\psi^{†}(r^{\prime },t^{\prime }\right){>}_{0eq}$$

where as before the terms *ψ*(r,*t*), *ψ*^†^(r,*t*) are annihilation, creation operators in the Heisenberg picture Equation (46) for the electron quantum fields, time-ordered by T_Keldysh_ (the Dyson time-ordering operator on the Keldysh contour). Equation (47) may be partitioned into four piece-wise defined Green functions chosen according to the location of the time variables *t*,*t*’ on the Keldysh contour. A common choice, which is adopted here, is closest to the Kadanoff-Baym definitions (a better choice for constructing equations of motion and self-energies is detailed by Lifshitz and Pitaevskii [70]):

(48) $$G_{}^{<}\left(r,r^{\prime },t,t^{\prime }\right)=G\left(r,r^{\prime },t,t^{\prime }\right)\;(t<t^{\prime })=i<\psi^{†}\left(r\prime ,t\prime )\;\psi^{\;}(r^{\;},t^{\;}\right){>}_{0eq}$$

(49) $$G_{}^{>}\left(r,r^{\prime },t,t^{\prime }\right)=G\left(r,r^{\prime },t,t^{\prime }\right)\;\;\;(t>t^{\prime })$$

(50) $$G_{\;}^{R}\left(r,r^{\prime },t,t^{\prime }\right)=-i\theta \left(t-t^{\prime }\right)<\{\psi^{†}\left(r{}^{\prime },t\prime ),\;\psi^{\;}(r^{\;},t^{\;}\right)\}{>}_{0eq}$$

(51) $$G_{\;}^{A}\left(r,r^{\prime },t,t^{\prime }\right)=i\theta \left(t\prime -t\right)<\{\psi^{\;}\left(r,t),\;\psi^{†}(r^{\prime \;},t^{\prime \;}\right)\}{>}_{0eq}$$

The Heisenberg equations of motion for these Green functions are coupled differential—integral equations that may be obtained by differentiating with respect to the two variables t and t’ (and r,r’). These equations immediately show up coupling to the two-electron Green functions that in turn are coupled to higher order Green functions to form a well-known hierarchy. Within systematic perturbation theory the hierarchy is curtailed by introducing self-energy terms that close off the hierarchy by simplifying the higher order Green functions. Under these conditions the equations of motion for the piece-wise time sub-domain Green functions are coupled but together form a closed system of equations. Using the Langreth theorem [40], which connects the Keldysh contour ordered Green functions to the real-time Green functions *G^<^*, *G^>^*, *G^R^*, *G^A^*, all the relevant Green functions satisfy a Dyson-like form with a suitable self-energy Σ(r,r’,*t*,*t*’):

(52) $$G^{\lambda }\left(r,r^{\prime },t,t^{\prime }\right)=G_{0}^{\lambda }\left(r,r^{\prime },t,t^{\prime }\right)+\;\iint^{\;}\iint^{\;}d^{3}r{}^{\prime \prime }d^{3}r{}^{\prime \prime \prime }d^{\;}t{}^{\prime \prime }d^{\;}t{}^{\prime \prime \prime }\{G_{0}^{\lambda }\left(r,r^{\prime \prime },t,t^{\prime \prime }\right)\Sigma \left(r{}^{\prime \prime },r^{\prime \prime \prime },t{}^{\prime \prime },t^{\prime \prime \prime }\right]G^{\lambda }\left(r{}^{\prime \prime \prime },r^{\prime },t{}^{\prime \prime \prime },t^{\prime }\right]\}$$

Here, $G_{0\;}^{\lambda }\;$is the relevant unperturbed Green function (*H*_int_ = 0).

Because the piece-wise Green functions are not independent, the self-energies are functionals of the other self-energies and Green functions. 

Considering now a non-equilibrium steady-state, energy-resolved picture let us transform to new variables τ*_relative_* = *t*–*t*’; *τ_mean_* = (*t* + *t*’)/2; then performing a Fourier transform of the Green functions over the relative time variable τ*_relative_* yields the same forms described in elementary fashion in Section 2.2:

(53) $$G^{<}\left(r,r{}^{\prime },\varepsilon \right)=\left(\frac{1}{\hbar }\right)\int_{-\infty }^{\infty }{d\tau }_{relative}\exp\left[\frac{i\varepsilon \tau_{relative}}{\hbar }\right]G^{<}\left(r,r{}^{\prime },\tau_{relative},\tau_{mean}\right)$$

(where there is no dependence on *τ_mean_* in the steady state) and similarly for *G*^>^, *G^A^*, *G^R^*. The Dyson Equation (52) become matrix equations in the space variables and the self-energies become convolution integrals over intermediate energies.

#### 2.3.2. The Steady State NEGF Equations

For the device Hamiltonian comprising an uncoupled term, an interaction term *H_int_* coupling electrons to phonons, and an applied perturbation *H_ext_* resulting from the applied electric fields and the confinement potential:

(54) $$H=H_{0}+H_{ext}+H_{int}$$

The resulting steady-state coupled Green function equations are:

(55) $$G^{R}={\left\{\varepsilon +i\eta -H_{0}-H_{ext}-\Sigma^{R}\right\}}^{-1}=G_{0}^{R}+G_{0}^{R}\Sigma^{R}G^{R}$$

(56) $$G^{A}=G^{R†}$$

(57) $$G^{<}=G^{R}\Sigma^{<}G^{A};\;G^{>}=G^{R}\Sigma^{>}G^{A}$$

Equation (55) is essentially a Dyson equation involving the self-energy $\Sigma^{R}$ which is a functional of the other Green functions and self-energies. Equation (57) is deceptively simple but it is the analogue of the classical Boltzmann equation as discussed briefly in Appendix C. Indeed from Equations (55) and (57) we have

(58) $$\{\varepsilon +i\eta -H_{0}-H_{ext}{\}G}^{<}=\Sigma^{R}G^{<}+\Sigma^{<}G^{A}$$

#### 2.3.3. The Projection Theorem

So far we have tacitly treated an open system. A key requirement in NEGF theory applied to device modelling is to project from the open system comprising a device coupled to its environment of contacts and thermal reservoirs into a finite simulation domain (see Appendix B, Figure A3). This non-trivial matter was first solved by Caroli et al in 1971 and their derivation of the theorem is sketched in the Appendix B. The main result is that the finite device domain may be modelled by the device Hamiltonian H_W_ (W for nanowire) provided that an additional set of non-Hermitian self-energies be added to H_W_ for each relevant Green function. These contact self-energies $\Sigma_{contacts}^{<},{\;\Sigma }_{contacts}^{>},\Sigma_{contacts}^{R},\Sigma_{contacts}^{A}\;$are localised interactions on each contact-device boundary surface that describe the injection or extraction of carriers (and consequently the inflow and outflow of charge, current and energy). 

#### 2.3.4. Numerical Solution of NEGF Equations

Equations (55)–(57) may be evaluated in the position representation, by inserting complete sets of position eigenstates; if the position variables are discretised the result is a set of matrix equations. The problem is significantly simplified if the self-energies are diagonal. We further require expressions for *H*_ext_ and the self-energies $\Sigma_{\;}^{\lambda },\Sigma_{contacts}^{\lambda }$.

In solving the NEGF equations, there are three other equations to be solved self-consistently: 

(i) The scattering self-energies must be evaluated self-consistently in at least the self-consistent Born approximation (fast algorithms for this and higher order perturbation are described in [75]. Appendix D gives an example of $\Sigma_{}^{<},{\;\Sigma }_{\;}^{R}\;$in the SCBA for a generic electron-phonon interaction. 

(ii) Poisson’s equation for the self-consistent applied potential and confinement potential (necessary for image charge induced electrostatic self-energies [10]) must be evaluated. 

(iii) If the lattice is considered to be held at constant ambient temperature, there is no further issue; if not, the heat equation(s) driven by the heat dissipation into the phonon system must be considered self-consistently. In the most advanced modelling, this latter step has been replaced by coupling the above equations self-consistently to the phonon Green functions [50,75]. A few groups use local atomic or molecular orbitals as the basis for tight binding style Hamiltonian models, but these techniques are restricted to a few hundred atoms [76]. The effective mass Hamiltonian method [76,77] is derived from tight binding and is discussed further in Section 3.

#### 2.3.5. NEGF Electro-Thermal Modelling

The NEGF equations are very powerful compared to Boltzmann transport theory because they are able to describe quantum phenomena such as tunnelling, resonances, bound states, fully quantised scattering theory, coherent and non-coherent flows. The density of states particularly LDOS can then reveal shift and broadening in the energy levels) due to the scattering self-energies/ contact self-energies) and hot spots in the spatially-resolved energy current densities. In the latter case we may immediately recover the quantum equivalent of the classical Equations (11) and (12):

The NEGF equivalents of the electro-thermally important quantities described in Section 2.1 are as follows.

The energy- and space- resolved charge density involves only the diagonal part of G^<^(r,r^′^,ε) (following from the limit (r→r^′^, *t*→*t*^′^), of Equations (47) and (48))

(59) $$n\left(r,\varepsilon \right)=-i\left(\frac{e}{2\pi }\right)G^{<}\left(r,r{}^{\prime },\varepsilon \right){|}_{r\prime \to r}$$

For a general Hamiltonian, *H* the velocity operator is defined in the Heisenberg picture by $v\equiv \mathrm{dr}/dt=\left(\frac{i}{\hbar }\right)\left[H,r\right]$.

For a simple effective mass Hamiltonian, the (electrical) energy-resolved current density is:

(60) $$J\left(r,\varepsilon \right)=ej\left(r,\varepsilon \right)={\left.\left(-e/4\pi m^{*}\right)\left(\nabla -\nabla^{\prime }\right)G^{<}\left(r,r^{\prime },\varepsilon \right)\right|}_{r^{\prime }\to r}$$

(61) $$J_{energy}\left(r\right)=\int \varepsilon \;j\left(r,\varepsilon \right)d\varepsilon \;$$

The local power transfer to the lattice is

(62) $$P(r)=\nabla \cdot J_{energy}\left(r\right)$$

or, separating out the Joule power,

(63) $$P(r)=-\nabla \cdot J_{kinetic}\left(r\right)+E\left(r\right)\cdot J\left(r\right)$$

where the first term on the RHS of Equation (63) is the divergence of the electron kinetic energy current density, while the second term is the product of the local electric field and the current density and represents the local Joule heat. The LHS is the net energy flow from the electron gas to the phonon assembly and is the source term for a heat equation (s). Equation (63) is derived by writing the kinetic energy $\varepsilon_{kinetic}\left(r,\varepsilon \right)=\varepsilon -e\phi \left(r\right)$. The kinetic energy current density is then

(64) $$J_{kinetic}\left(r\right)=\int \varepsilon_{kinetic}\;j\left(r,\varepsilon \right)d\varepsilon \;$$

and

(65) $$J_{Energy}\left(r\right)=J_{kinetic}\left(r\right)+\phi \left(r\right)J\left(r\right)$$

Taking the divergence of Equation (65) and using E(r)=−∇ϕ(r) plus the continuity equation result ∇·J(r)=0, recovers Equation (63).

Using Equations (55)–(57), we obtain an expression for the divergence of the total energy current density

(66) $$\nabla \cdot J_{Energy}\left(r\right)=\int d\varepsilon \;\varepsilon \int d^{3}r^{\prime }\{\{G^{R}\left(r,\;r\;{}^{\prime },\varepsilon \right)-G^{A}\left(r,\;r\;{}^{\prime },\varepsilon \right)\}\Sigma^{<}\left(\;r\;{}^{\prime },r^{\;},\varepsilon \right)+\;{\;G}^{<}\left(r,\;r\;{}^{\prime },\varepsilon \right)\Sigma^{A}\left(r{}^{\prime },r^{\;},\varepsilon \right)-\Sigma^{R}\left(r,\;r\;{}^{\prime },\varepsilon \right)G^{<}\left(r{}^{\prime },r^{\;},\varepsilon \right)\}$$

(67) $$\nabla \cdot J_{Energy}\left(r\right)=\int d\varepsilon \;\varepsilon <r|\{G^{R}\left(\varepsilon \right)-G^{A}\left(\varepsilon \right)\}\Sigma^{<}\left(\varepsilon \right)+G^{<}\left(\varepsilon \right)\Sigma^{A}\left(\varepsilon \right)-\Sigma^{R}\left(\varepsilon \right)G^{<}\left(\varepsilon \right)|r>$$

The operator within the position matrix element of Equation (67) is related to the current operator as discussed in the Appendix D, Appendix E and Appendix F.

Since the self-energies here include coupling to the contacts it is possible to use Equation (66) to locate precisely where the energy dissipation occurs (for example, within particular device regions or at the contacts). 

The above equations plus explicit forms for the electron-phonon interaction self-energies (see Appendix D) give the quantum equivalent of the classical electro-thermal formulation of Section 2.1, i.e., Equations (11) and (13). It is important to demonstrate that expression $\nabla \cdot J_{Energy}\left(r\right)\;$vanishes for elastic scattering. This is demonstrated in Appendix E, where $\nabla \cdot J_{Energy}\left(r\right)\;$is shown explicitly to vanish everywhere within the device volume using the self-energies for high temperature elastic acoustic phonon scattering in the self-consistent Born approximation. For the elastic case, there is no energy dissipation within the channel but when the contact self-energies are introduced there may still be energy dissipation in the drain contact region. 

## 3. Simulation Methodology for the Silicon Gate-All-Around Nanowire FET

In this section we describe the carrier transport model and our electrothermal model used in the simulation of a gate-all-around nanowire transistor. In a nutshell, our method simultaneously solves the NEGF Equations (55)–(57), the Poisson Equation (3) and heat Equation (15) with the appropriate boundary conditions.

We start by estimating the electrostatic potential and use it to solve the NEGF Equations (55)–(57) for a range of energies; these two equations need to be solved self-consistently as electron-phonon scattering self-energies depend on the electron density. From the calculated Green functions, the electron density is obtained and inserted into the Poisson equation to calculate the new electrostatic potential. At the same time, the current and Green functions are used to calculate the local power dissipation that is the input into to the heat equation in order to find the temperature profile. This process continues until density, current and temperature do not change. In what follows, we will describe in detail the different physical models, boundary conditions and simplifications used in solving the equations.

### 3.1. Device Structure and the 1D Representation

**Figure A2:**
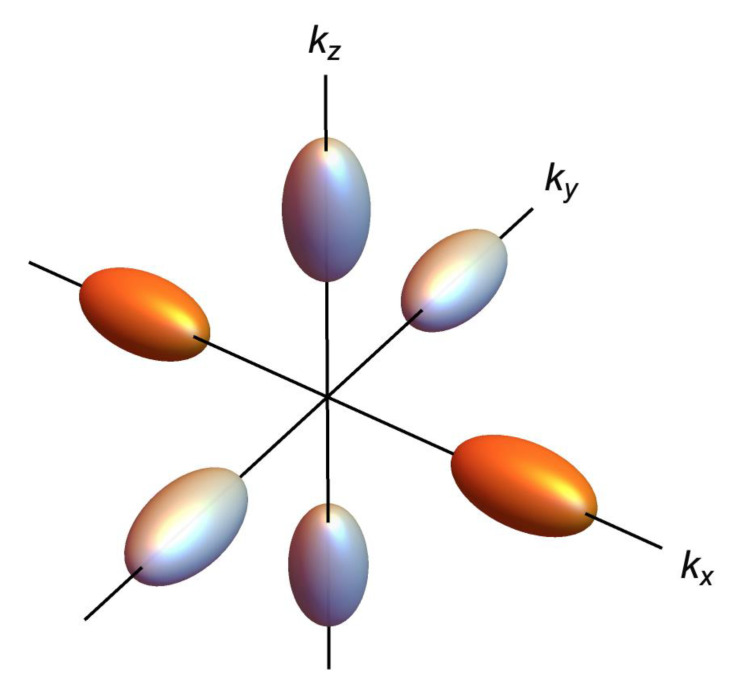
Constant energy surfaces for the Δ valleys in the k-space used to represent the effective mass Hamiltonian for a silicon nanowire device. The principal valleys for transport are shown in orange. In our work the *k_x_* aligns with the channel of the wire.

**Figure A3:**
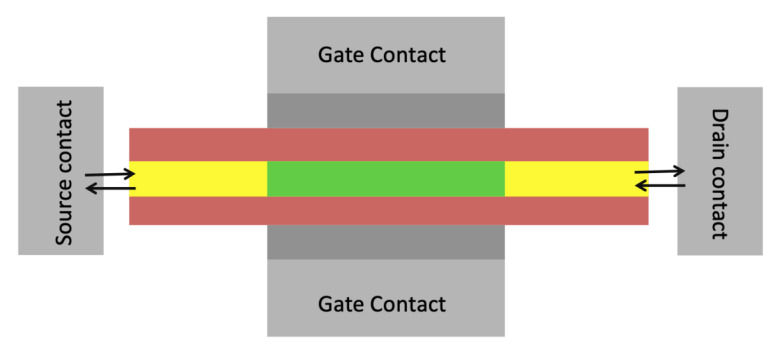
Simplified plan of simulation domain (in yellow-source extension, green-channel, brown-oxide, dark grey-wrap-round gate) and macroscopic environment /reservoirs (light grey-macroscopic contact domains). No channel: gate current.

In this work, the effective mass approximation [78,79,80] has been used to approximate the non-interacting Hamiltonian *H_0_* of the silicon crystal entering in Equation (54). The effective masses used have been extracted from tight-binding calculations, see reference [80]. In addition, our model for Si considers anisotropic bands and includes the six Δ-valleys, see Appendix A and Figure A2 for the *k*-space location of the valleys. The axis of the wire is oriented in the [100] direction, for a square cross section, 4 ellipsoids are equivalent as shown in Figure A2. 

In devices with cylindrical or rectangular symmetry the spatial dependence of the Green functions may be reduced to 1D forms (in, *x,x*’) by projecting out the transverse (*y,z*) dimensional dependence using self-consistent transverse eigenstates (discrete sub-bands *n*) computed at each point along the *x*-direction (see Section 2.3 and references therein). The full Green functions are then replaced by effective 1D Green functions: $G\left(x,x^{\prime },\varepsilon \right).$ This simplification is a key result for the efficient modelling of nanowire structures. 

**Figure 2:**
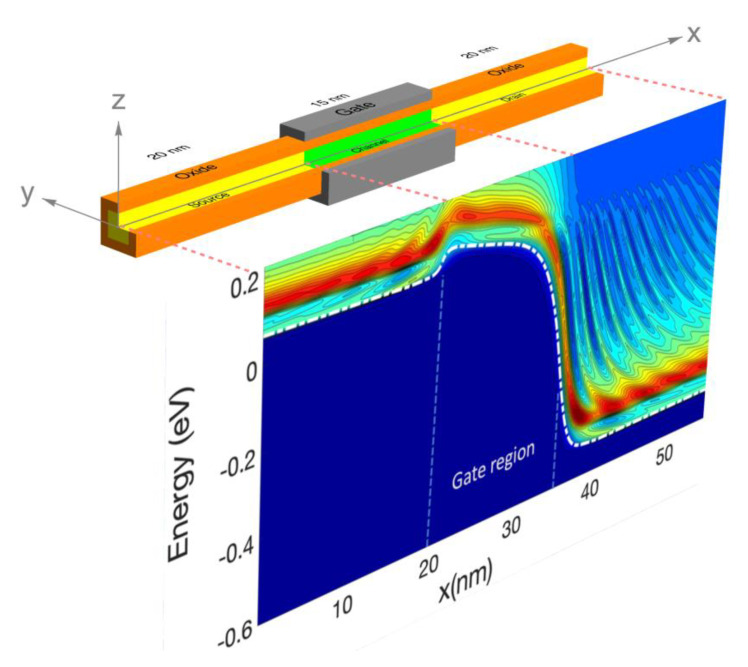
Schematics of a wrap-round gate silicon nanowire field-effect transistor of length 55 nm cut away to expose the channel region. The oxide thickness is 0.8 nm and the channel width is 2.2 nm. The channel length (gate length) is 15 nm, the source and drain extensions are 20 nm. The work function of the metal gate is 4.5 eV. The projected image shows a typical non-equillibrium Green function (NEGF)-computed local density of states (LDOS) for the device assuming a drain bias of 0.6V and a gate bias at 0.6 V. The location of the first 1D sub-band edge is shown along the central nanowire x-axis as a function of energy and position (dashed white line); it mimics the self-consistent potential along the same axis. In the projected image, the vertical energy scale is from −0.6 eV to 0.23 eV.

The device (typified by Figure 2) is divided in cross-sections perpendicular to the axis of the wire. The standard Schrödinger equation is solved in each cross-section by using the electrostatic potential calculated by the Poisson equation. This results in a set of eigenstates for each valley and cross-section considered. These eigenstates are commonly called sub-bands or modes and are associated with individual conduction band valleys. The longitudinal Hamiltonian is expanded in these sub-band states that are coupled to each other by the relevant overlap integrals. This method is called the coupled mode space method [81,82,83,84] and is very efficient for very narrow nanowires as the number of sub-bands considered can be kept to a minimum. In detail, the full volume of the device simulation domain is discretised in a rectangular mesh; the Schrödinger equation is then solved for each cross-section perpendicular to the wire axis. The potential energy entering in the equation is the electrostatic potential energy calculated from the Poisson equation and the confining potential at the SiO_2_ interface. The kinetic energy is given by the anisotropic mass tensor corresponding to the particular valley. From the solution of the Schrödinger equation, a set of eigenvalues and eigenvectors (wavefunctions) are obtained for each cross section. These eigenvectors or modes are used to expand the 3D spatially discretized Green function [84] of size (n_x_ × n_y_ × n_z_, n_x_ × n_y_ × n_z_), where n_y_ and n_z_ is the number of discretisation points in the cross-section direction and n_x_ in the transport direction. From these modes and by integration in the transversal spatial coordinates, an equation for the Green function is constructed that couples the modes in one cross section with the modes of the nearest neighbouring cross-sections [83,84]. The size of this Green function matrix is of order (*n_m_* × *n_x_*, *n_m_* × *n_x_*), where *n_m_* is the total number of modes used in the simulations. Note that the 3D spatially resolved Green function is substantially larger in size than the mode space Green function as mentioned before. In the discretised Hamiltonian, the matrix element (or coupling) between mode *m* in cross section *i* and mode *l* in cross section *i + 1* is given by:

(68) $$-\frac{\hbar^{2}}{2m_{x}{\left(\Delta x\right)}^{2}}\iint \psi_{i}^{*m}(y,z)\psi_{i+1}^{l}\left(y,z\right)dydz$$

where $\psi_{i}^{m}\left(y,z\right)$ is the wavefunction for mode *m* in cross section *i*, the *y* and *z* are the spatial coordinates representing the cross section. Δx is the discretisation step in the longitudinal direction or the transport direction. The asterisk * in the above expression stands for the complex conjugate. The double integral is over the whole cross-section. Equation (68) shows that the probability of an electron going from mode *m* in cross section *i* to mode *l* in cross-section *i + 1* depends on the overlap between wavefunctions. It should be noted that if the potential energy in the cross-sections are similar the above integral between two different modes is zero. This means that there is a very small probability (or amplitude) for the electrons during their motion along the wire to switch modes. When the integral between different modes is assumed zero, the simulation method is called the uncoupled mode space approach. In this case the Green function in the mode/longitudinal space reduces to n_m_ Green functions of size *(n_x_* × *n_x_)*. This reduces the computational load substantially. After the longitudinal Green function is obtained for each valley, mode and energy, the electron density and current is calculated from them and the mode wavefunctions. For details of the coupled mode space as applied to NEGF, we recommend the following references [83,84]. 

We have considered the acoustic and optical (for f-type and g-type) intervalley scattering mechanisms, as well as the intra-valley elastic acoustic phonons scattering mechanism. The specific parameters and models used can be found in references [85,86]. The electrostatic potential is calculated from the Poisson equation in the mean field approximation. We have used Neumann boundary conditions in the source and drain contacts; and Dirichlet boundary conditions at the gate contact.

Figure 2 shows the schematics of a typical wrap-round gate silicon nanowire with a silicon core comprising source, channel and drain regions. There is a high degree of symmetry. Therefore, we may use the sub-band projection methodology to obtain the 1D density of states throughout the silicon core as a function of energy and position as indicated.

**Figure 3:**
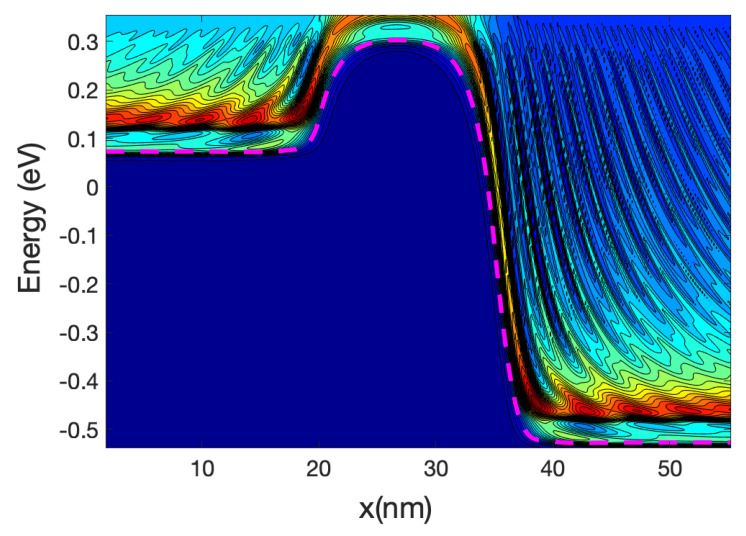
Local density of states (LDOS) along the nanowire axis. The dashed line depicts the first sub-band edge. Dark red indicates high LDOS and dark blue low LDOS. The drain bias is 0.6 V and the gate bias is 0.2 V.

Figure 3 shows the detailed 1D local density of states (LDOS) computed along the nanowire axis for each sub-band energy: it is proportional to (*ε−ε_n_*)^−1/2^ where *ε_n_* is the local sub-band energy. Notably, the multiple reflection patterns (a simple example is in Appendix A) that are shown for energies lower that the top of the maximum sub-band energy around the middle of the channel. This is an indication of coherent transport. The wavelength of the electrons increases as the energy increases; this can be confirmed by the shorter separation of the maxima and minima in the figure. 

### 3.2. Non Equilibrium Green Function Formalism: Density and Current Density

Equations (41) and (42), used for the calculation of the Green function, implicitly contain Pauli’s exclusion principle [57,58,87]. There, $\Sigma^{<}$ is a sum of two terms, one is proportional to the electron-phonon scattering rate and the other representing the boundary self-energies that are different from zero only at the contacts. This latter term involves the product of the electron distribution of the contact and the density of states at the contact. This approximation in the contacts is a consequence of the fluctuation-dissipation theorem [88]. In one dimension, for each mode, the energy-resolved electron concentration and current density could be calculated from the mode-space Green functions by [83,84]:

(69) $$n_{l}\left(x,\varepsilon \right)=-\left(\frac{i}{2\pi }\right)G_{ll}^{<}\left(x,x,\varepsilon \right)$$

(70) $$J_{nl}\left(x,\varepsilon \right)=-\frac{e}{4\pi m^{*}}\underset{x^{\prime }\to x}{\lim}\;\left(\frac{\partial }{\partial x}-\frac{\partial }{\partial x^{\prime }}\right)G_{nl}^{<}\left(x,x^{\prime },\varepsilon \right)$$

where *x* represents the spatial coordinate, e the electron charge snd m* the effective mass. The *n* and *l* in the RHS label the modes. Equations (69) and (70) must be modified by a factor of two for spin degeneracy. The total 3D electron density is calculated from the above 1D electron density and the cross-section wave function by:

(71) $$n\left(r,\varepsilon \right)=\underset{l}{\sum }n_{l}\left(x,\varepsilon \right)\psi_{x}^{*l}\left(y,z\right)\psi_{x}^{l}\left(y,z\right)$$

**Figure 4:**
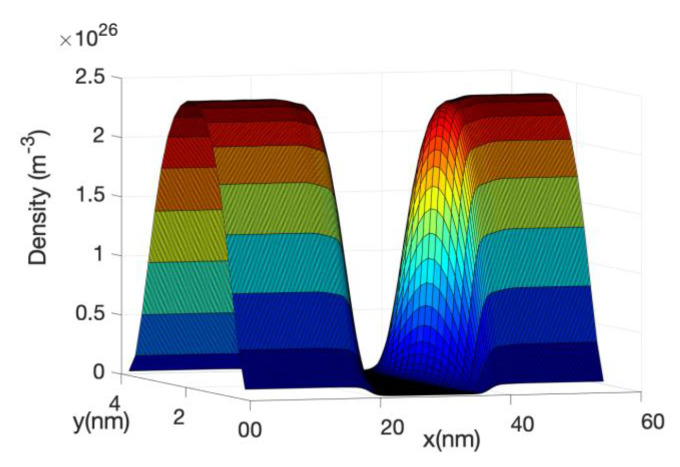
The electron density along the nanowire axis in a parallel xy plane at Vg = 0.4 V and Vd = 0.6 V. The figure shows the volume inversion and also the fulfilment of charge neutrality, see text.

Figure 4 shows the electron density at low and high gate bias, showing electrical neutrality, i.e., the potential is flat at the contact and the electron concentrations are similar in the source and drain contact. The electron density drops close to the oxide interface showing the volume inversion and the shape of the ground state transversal wave function. For narrow-body nanowires, this concentration of electrons along the wire axis is usually called the volume inversion or electron confinement. At very high gate voltage, the source-drain barrier disappears and, in order to fulfil electrical neutrality, the leads or ends of the source and drain region should be broadened (see Landauer [59]). At high gate bias, the electron potential energy along the wire direction resembles a step function or a linear drop with an energy difference proportional to the applied bias, so the right moving waves from the source reach the drain but the left moving waves from the drain are reflected by the energy drop. This means that the density of electrons in the drain will be always larger than in the source and therefore electrical neutrality would not be fulfilled. However, if simulations are carried out with broadened leads, electrical neutrality in the source/drain are independent of the gate potential and are controlled by the electrostatic of the leads.

**Figure 5:**
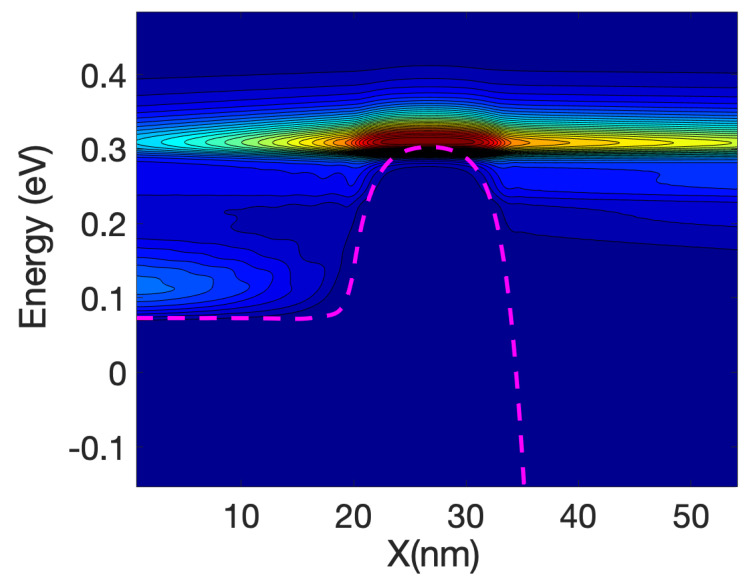
Energy resolved current spectra along the wire axis. The first sub-band is shown as a dashed line. The current at the source (in the left side of the figure) divides into two branches around energies 0.1 eV and 0.3 eV. The current in the low energy branch disappears when progressing from the source towards the right, vice-versa the current in the high energy branch increases. These means that the electrons from the lower energy branch are moving to the right branch. These electrons need to absorb phonons to increase their energies and therefore they tend to cool the lattice at the source.

Figure 5 shows the energy resolved current spectra calculated by Equations (69) and (70) along the nanowire transistor axis. The source contact is in the left end and the drain is at the right end. The first sub-band is shown as reference. The part of the current with energy lower than the top of the barrier sub-band energy represents tunneling current. As the device has a 15 nm channel length, the tunneling current is not negligible. From the figure it can be seen that electron at the source moving from the source towards the drain comes from two different energy regions:

(i) energy close to the top of the barrier and
(ii) energy close to the sub-band energy at the source contact.

Electrons entering the source region (i) will be able to go through the barrier, unless they emit phonons and lose energy. Those in region (ii) need to absorb phonons in order to cross the barrier and in doing so will cool the lattice locally. The temperature profile simulations presented in the next section confirm this statement. At the drain side, electrons injected from the source (at high energy) start to dissipate part of the energy and the current at the drain is more spread downwards in energy compared with the current at the top of the barrier. However, the average energy of the electron at the drain end is approximately equal to 0.25 eV that is substantially larger than the sub-band energy (−0.55 eV).

### 3.3. Energy Balance and Power

The Equations (61)–(63) represent the energy balance between electrons and phonons and is discussed in several references [89,90,91,92]. 

These equations represent the total local power dissipated for the electron system into the phonon system. As indicated before, the last term on the RHS of Equation (63) is the local Joule power that integrated through the device produces the well-known Joule power *I·V*. In Equation (63) the term E(r) represents the local electric field along the nanowire axis. The first term on the RHS is the change in the “kinetic energy” current of the electron ensemble, if this kinetic energy current does not change it means that the electron system is adiabatically following the “bending” of the potential and therefore it is not increasing the electron mean energy relative to the conduction band minima, i.e., no hot electron phenomena occur. In this case, the electron system or more specifically the electrons taking part in the current are in equilibrium with the local potential. On the other hand, a large change in kinetic energy current implies the heating or cooling of the electron system. 

Equation (63), for the local power, can be simplified for the case of local electron-phonon self-energies and the resulting equation obtained [56] is essentially a detailed balance equation (see Appendix E, F specificaly Equation (A46)):

(72) $$P\left(r\right)=\int d\varepsilon \;\varepsilon \left\{G^{<}\left(r,\;r,\varepsilon \right)\Sigma^{>}\left(r,\;r,\varepsilon \right)-G^{>}\left(r,\;r,\varepsilon \right)\Sigma^{<}\left(r,\;r,\varepsilon \right)\right\}$$

The electron-phonon self-energies used in this paper are also treated in a local approximation (diagonal approximation), so that Equation (72) is the local version of Equation (A46) in the Appendix F. The RHS of Equation (72) expresses the local power as a function of the Green functions and self-energies. It shows that the energy mismatch between the rate of ε -outgoing (leaving the state of energy ε) electrons (the first term in the RHS) and the rate of ε -outgoing holes (second term in the RHS) is transferred to the phonon system. Note that for elastic scattering the expression in the curly brackets became zero [91] (Appendix E demonstrates this explicitly). As mentioned before, the first term inside the integral in the RHS represents the outgoing electrons from state ε and the second term is the ingoing electrons into state ε. For elastic scattering these rates are equal as the electron does not change its energy after scattering and *P(r)* vanishes except in the contacts. Equation (72) represents the detailed energy balance between the electron and the phonon system.

**Figure 6:**
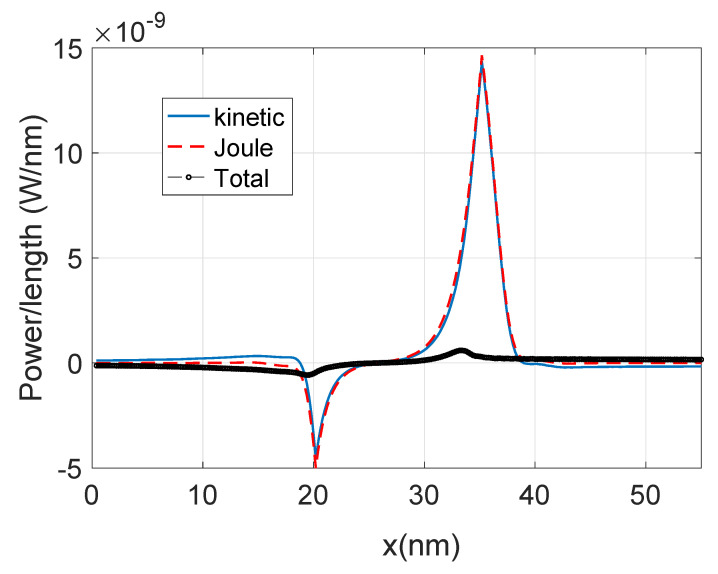
Local power dissipated along the device axis. Vg = 0.4 eV. The thin line and the dashed line represent the corresponding values of the kinetic term and the Joule term in Equation (48) of the text. The thick line represent the total power (the subtraction of the two graph). It should noted that the actual power dissipated inside the device (signed area under the curve) is much smaller than the Joule power (Joule term).

Figure 6 shows the kinetic term and the Joule term of Equation (63). Their sum or the total power is also shown. Note that the kinetic term is plotted with reverse sign. The two terms are very similar but with opposite sign therefore in order to obtain an accurate value of the total power, the current needs to be calculated with great accuracy which implies a large number of Born iterations for the self-energies.

The 3D Fourier heat equation used to calculate the local temperature profile is solved in the oxide and in the Si nanowire core. Neumann boundary conditions are used in the source and drain contacts and the Dirichlet boundary condition is used at the metal gate. The heat sources are calculated from the total power or using the Joule heat power, depending of with model we are using. Both sources include the volume inversion effects. 

### 3.4. Results and Discussion

In this section, we report electrothermal simulations of a gate all around nanowire transistors (Figure 2). The cross section of the silicon core is 2.2 × 2.2 nm^2^ and the source, channel and drain have lengths of 20 nm, 15 nm and 20 nm, respectively. The channel is undoped and the doping in the source and the drain are 10^20^ cm^−3^. In all of this work, we assumed a drain bias of V_d_ = 0.6 V. We have compared the drain current vs gate voltage characteristic (transfer characteristic) for the case of standard scattering, i.e., assuming the phonon system or the lattice is at equilibrium at 300 K with cases in which the lattice is at higher temperature. 

The local phonon temperatures are calculated from Fourier’s law. Two cases have been considered: case (i): in which the electrons are in local equilibrium with the electrostatic potential, so the energy transfer from the electron system to the phonon system is given by the standard Joule law or the second term in Equation (63); and case (ii), in which the electrons relax to equilibrium depending on the energy relaxation length (given by the inelastic electron-phonon scattering length) [92] and therefore the energy transfer are given by the LHS of Equation (63). This latter case considers that the electrons injected from the source into the drain leave the drain at a higher energy than the drain average (equilibrium) energy. These electrons are commonly called “hot electrons”. Consequently, these electrons dissipate less energy inside the device as compared with the electrons in local equilibrium and therefore produced less heating of the lattice in the active part of the device. 

In a previous paper [92], we have simulated and examined different sizes of source and drain extensions for a nano-transistor with a similar cross section to the one studied here but self-heating was not considered. In that paper, we found that electrons needed a drain extension length larger than 100 nm in order to guarantee that the total power dissipated inside the device became equal to the Joule power. 

However, even for larger source and drain extensions as long as the channel length is around 10 nm, inside the device, the potential energy of the electrons changes hundreds of millielectron volts in just a few nanometers. This length is too short compared with the energy relaxation length. As a consequence, the electron transport around the gate is essentially out of equilibrium or carried out by “hot electrons”. The energy relaxation depends on the density of states and on the electron-phonon scattering rates. However, previous papers [76] for small cross section devices confirm an increase in electron-phonon scattering rates due to an increase in scattering form factors. This fact favours a decrease in the energy relaxation length in small devices but this is not sufficient to allow full electron energy relaxation inside the device.

In general, devices with cross sections larger than 10 × 10 nm^2^ and with channel/source/drain of a few 100 of nanometers are expected to comply with the Joule law but as mentioned previously a more accurate assessment would look at the local spatial variation in the electrostatic potential or electron potential energy, which depends on the device structure and dimensions.

**Figure 7:**
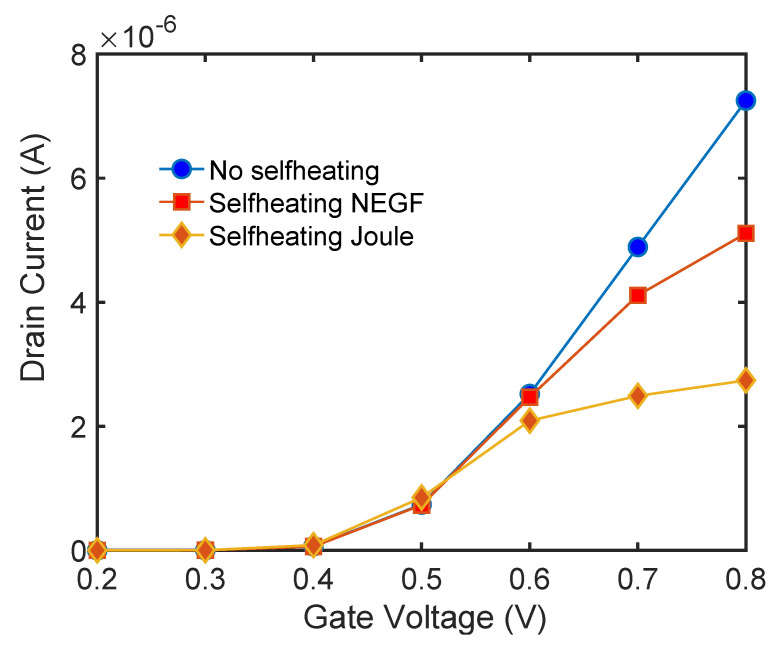
Transfer characteristic for cases (i) no self-heating (NSH), (ii) hot electrons self-heating (HSH) and (iii) Joule self-heating (JSH). In the no self-heating case the lattice is keeped at 300 K. In the other cases the local lattice temperature depend on the net power dissipated by the electron system locally to the phonon system. For the case (iii), the electron system is considered at local equilibrium with the lattice. The drain bias is Vd = 0.6 V. At high gate bias, the current for the of Joule selfheating is larger than the current for the hot electron one, this is a consequence of the higher temperature of the lattice for the joule case as compared to the hot electron case.

The current-voltage characteristic for these three scattering simulation cases: (i) no self-heating (NSH), (ii) hot electrons self-heating (HSH) and (iii) Joule self-heating (JSH) are shown in Figure 7. 

As it is clear from Figure 7 that under self-heating conditions, i.e., in which the lattice has a high temperature, the scattering rate is large and the current at Vg = 0.8 V is 30% smaller for the hot electrons case as compared with the non-self heating case. However, when the Joule self-heating is considered the effect is larger, and the drain current is reduced by 70%, compared to the NSH case. For low gate bias, as the current is small, the impact of self-heating and scattering in general is diminished. In what follows, we will discuss the results at low gate bias and large gate bias. 

**Figure 8:**
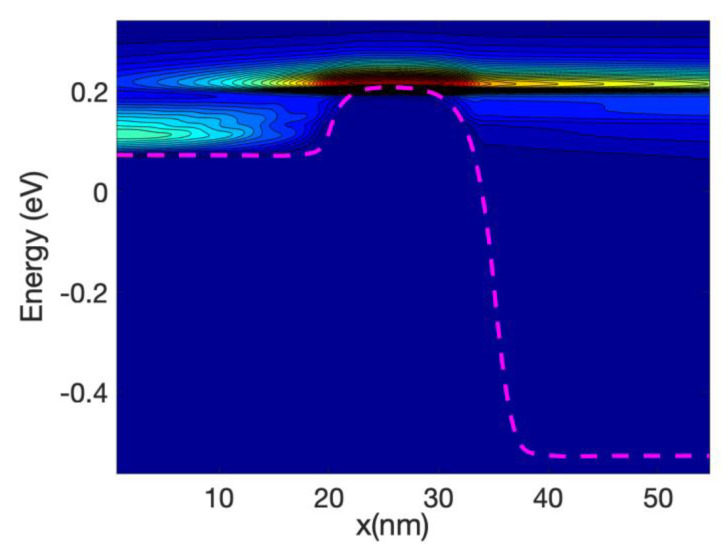
Energy-resolved current along the nanowire axis at Vg = 0.2 V. The first sub-band is shown by a dashed line. The electrons contributing to the current at the source (leftmost part in the figure) have lower energies than those at the middle of the device (or at the barrier top). Indicating large phonon absorption at the source (see the text).

**Figure 9:**
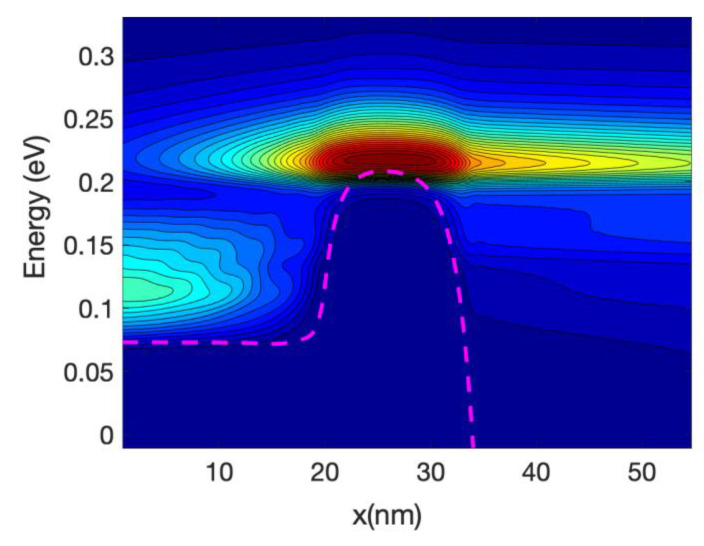
Close-up of Figure 8. The current spread in energy is very small at the middle of the wire or at the top of the source drain barrier. However, the spread in current increases as electrons travel towards the drain contact indicating energy relaxation. The average energy of the current is around 0.2 eV at the drain contact.

At low gate bias the current is low and consequently the power dissipated is low; however, the fact that we have a large source-drain bias barrier produce some interesting effects. Figure 8 and Figure 9 show the current spectra at Vg = 0.2 V for the case of the hot electrons. Figure 9 shows an enlargement of the high energy region of Figure 8 where the electrons energy exchange occurs.

The figures show that the electrons at the bottom of the band entering in the source absorb phonons and therefore are able to cross the barrier, this phonon mediated effect tends to transfer energy from the source to the drain of the device, and therefore it has a cooling effect on the source of the device and slight heating at the drain. 

As the nanowire cross-section is small the LDOS is quasi-1D and therefore there is a relatively large DOS at the sub-band energy at the source end (and indeed at the drain end); consequently, there is an overwhelming number of electrons at that low energy. This induced a high probability of phonon absorption for the electrons in the source end. This is one of the reasons that the energy-resolved current at the source is split into two components for a large source-drain barrier.

**Figure 10:**
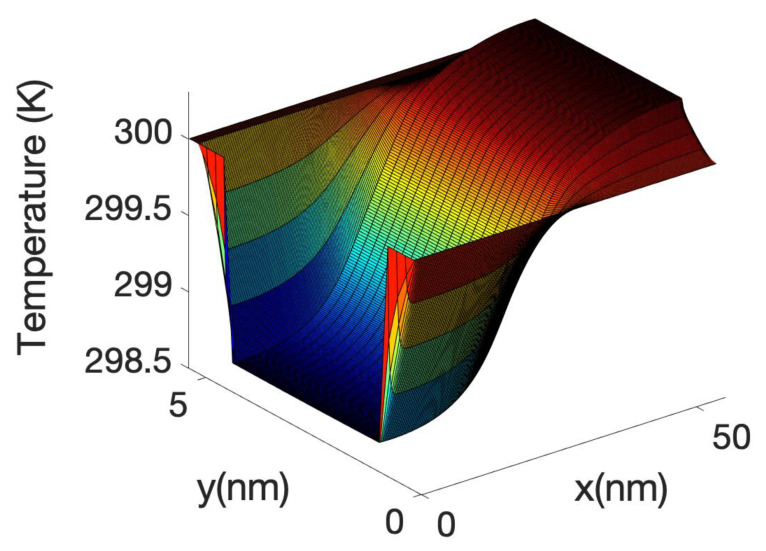
Temperature profile for (x,y) plane for the z coordinate located at the middle of wire cross section; for the coordinate system used and the device orientation see Figure 2. The profile corresponds to the hot electron self heating case (HSH) at a low bias of Vg = 0.3 V. The figure shows a slight cooling of the source of the transistor. The overall power dissipated inside the transistor is negative.

**Figure 11:**
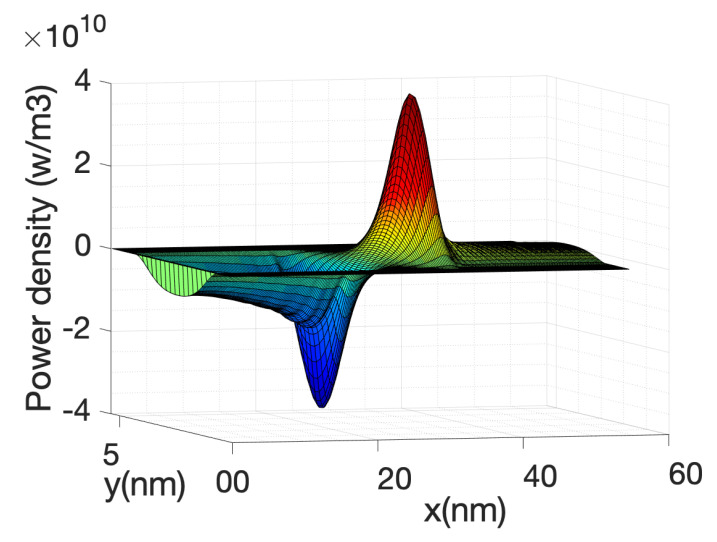
Power density profile used as heat source in the heat equation.The gate potential (Vg) and drain potential (Vd) are 0.3V and 0.6 V, respectively. This figure shows the 2D profile of the 3D analogue of the total power showed in Figure 6. Positive values indicate source of heat and negative values sink of heat or cooling.

Figure 10 shows the temperature profile across a plane of the device axis including the oxide at Vg = 0.3 V. The figure shows a slight cooling (from 300 K) of the source of the device. This effect is slightly enhanced at Vg = 0.3 V as the height of the barrier has decreased and therefore the electrons are more efficiently transferred lower energy in the source to the top of the barrier. The cooling of the source diminished at higher gate bias as a large fraction of electrons are able to tunnel or directly transit the barrier without the need of phonon assistance. This indicated an optimum length in which the cooling of the source is more efficient. At very low gate bias we have found that the total power dissipated by the electron current in the device is negative; this means that the electrons absorbing energy from the phonon system on the source are not able to release enough energy at the drain to counteract the cooling effect at the source. This net energy absorption of electrons in the source region has been observed in other papers [86,91] through the calculation of the power absorbed along the wire for the electrons using the Equation (9) as will be shown in the Figure 11, here. As mentioned before, it can also be inferred by the plot of the energy current resolved along the nanowire axis (see Figure 8 and Figure 9). However, Figure 10 shows a decrease in temperature (cooling) of the lattice, which depends on the net interchange of heat of the lattice with the surrounding, as it is not sufficient that the electron system absorb energy from the lattice locally in order to cool the lattice. If the drain or other parts are hot enough, heat will be transfer to the source avoiding cooling. The fact that the electron going through the drain does not release enough energy there is one of the facts which allows for the cooling of the source. If we had assumed that the electrons are in equilibrium with the potential or, i.e., assuming Joule heating, the net power dissipation would be always positive, that is a power transfer from the electrons to the lattice. In addition, there would have been a large dissipation in the drain, heating the overall drain and surroundings including the source.

The local power density profile used as a source in the heat source is shown in Figure 10 along the nanowire axis for the case of hot electrons. It is shown that most of the power transfer occurs close to the source-drain barrier. The negative power density at the source end of the barrier indicates that the electron system is taking energy from the lattice or phonon system as described before.

**Figure 12:**
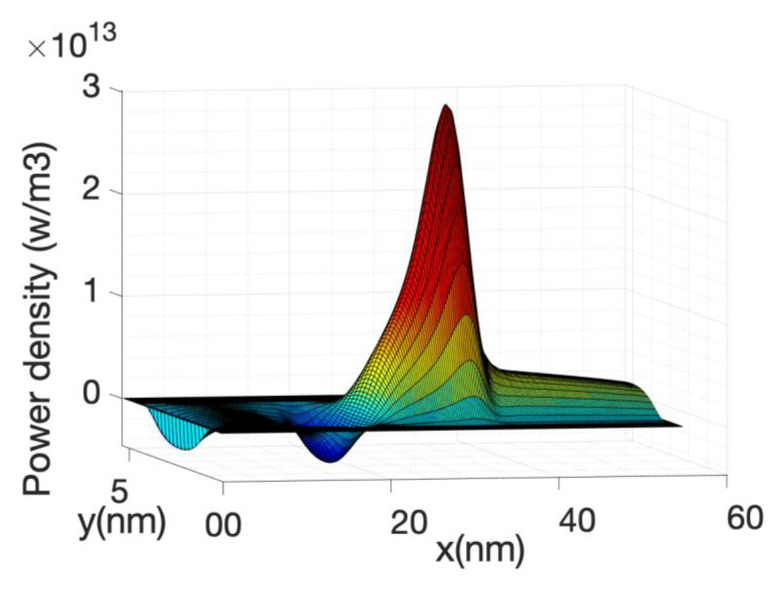
Heat source due to hot electrons at Vg = 0.6 V.

**Figure 13:**
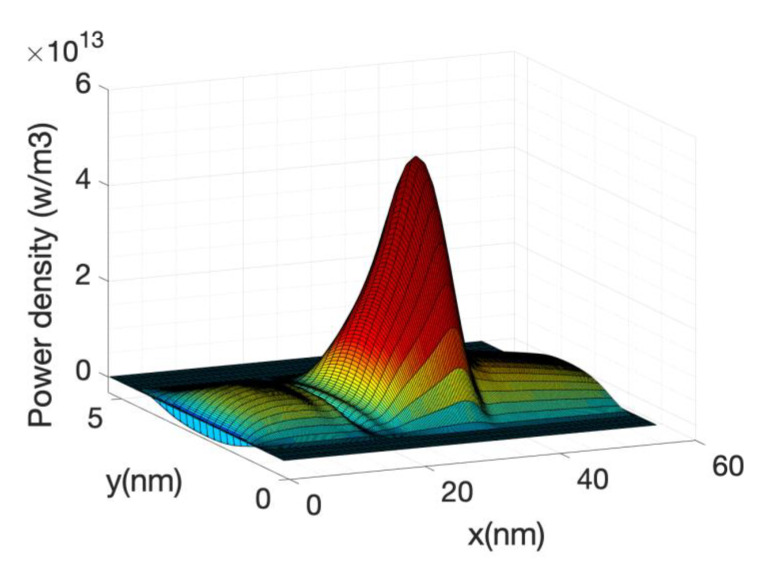
Heat source due to hot electrons system at Vg = 0.7 V.

**Figure 14:**
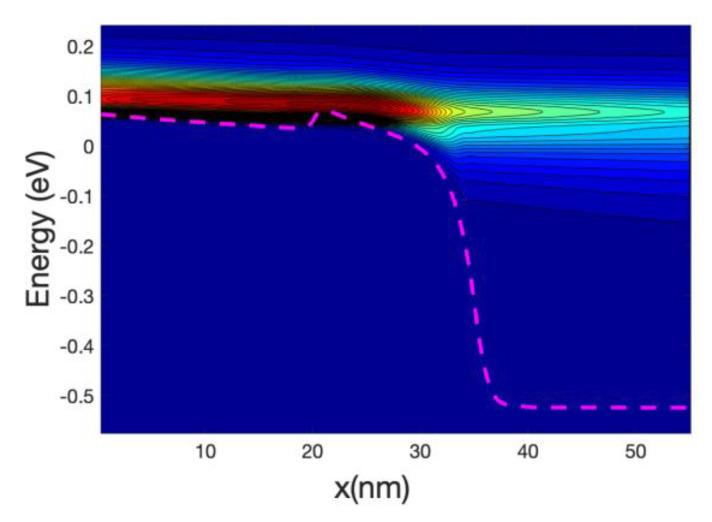
Energy resolved current spectra Vg = 0.7 V, hot electrons and selfheating.

**Figure 15:**
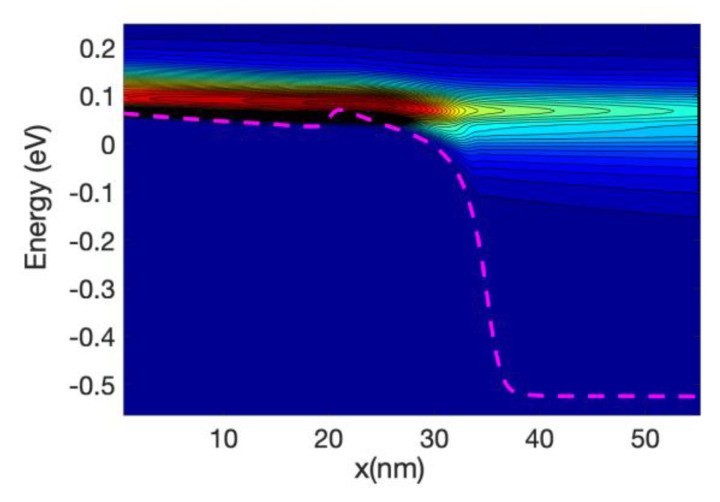
Energy resolved current spectra Vg = 0.6 V, hot electron source and self-heating.

**Figure 16:**
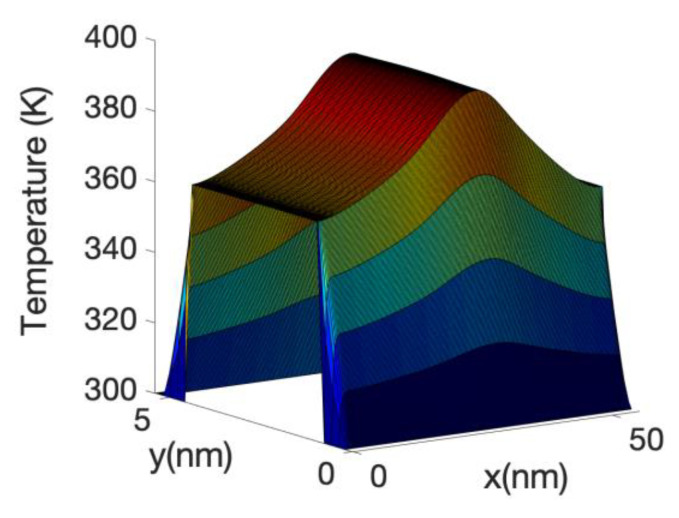
Lattice temperature profile at Vg = 0.7 V, hot electrons and self-heating.

**Figure 17:**
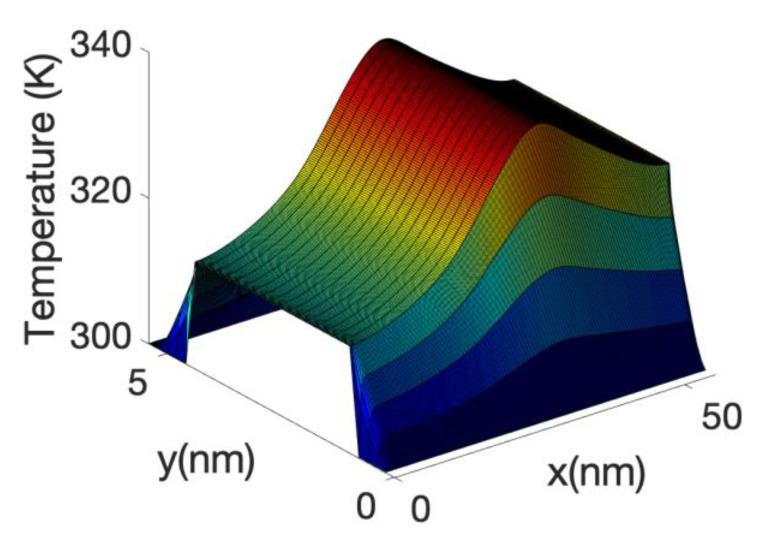
Lattice temperature profile at Vg = 0.6 V, hot electron source and self-heating.

At high gate voltage, the source-drain barrier is small or negligible. Figure 12 and Figure 13 show the profiles of the heat source term for Vg equal to 0.6 V and 0.7 V, respectively. At Vg = 0.6 V, there is a small region in which the heat source has negative values: in this region the electrons are still absorbing energy from the lattice; however, there is a greater heat transfer from the electrons to the lattice at the drain region and as a consequence the overall temperature increases. When the barrier is sufficiently low, as is in the case of Vg = 0.7 V (Figure 13), there is no region of negative-valued heat source; in this case, the potential resembles a step potential and the electrons just release energy when transiting the device. The energy-resolved current density for Vg = 0.6 V and 0.7 V is shown in Figure 14 and Figure 15, for the case when self-heating and hot electron effects are considered. The corresponding temperature profiles are shown in Figure 16 and Figure 17. Note that although the current spectra look very similar, the change in maximum temperature is approximate 60 K. Figure 17 resembles the 1D temperature plots of Figure 7 in reference [93]. We have carried out a more thorough comparison in our previous paper [94]. However, it is worth mention that our nanowire has rectangular cross section and the nanowire in [93] has a circular cross section (not exactly matching the areas). We are using an effective mass approximation, and the reference [93] used full band. The scattering models are also different as they used the full phonon bands. The reference [93] simulated the phonons using a NEGF formalism instead of our simplified heat equation. However, the level of current obtained in our simulations are of similar order of magnitude to those in [93]. Considering all the differences between the models, our calculated temperature profiles qualitatively follow those found in [93]. The temperature profiles there range from 300–500 K and this also matches the range of our results. 

**Figure 18:**
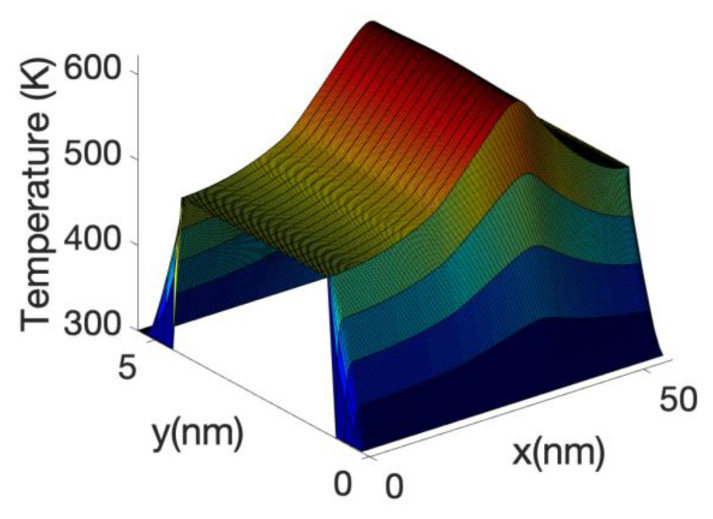
Lattice temperature profile for Vg = 0.6 V considering Joule self-heating. This figure is to be compared with figure 17. The peak temperature is almost twice the value of Figure 17. Indicating an overestimation of power dissipation.

**Figure 19:**
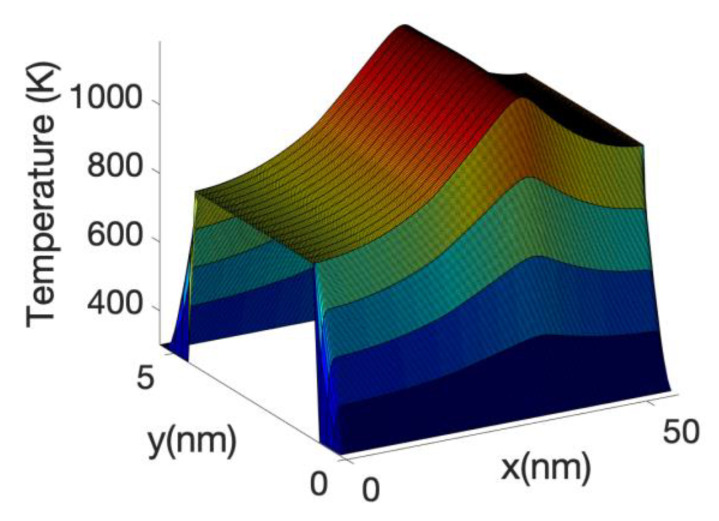
Lattice temperature profile for Vg = 0.6 V considering Joule heat source but no self-heating. The temperature peak is large than 1000 K. This is a substantial overestimation of lattice heating when compare with the more accurate hot electron heating of Figure 17.

**Figure 20:**
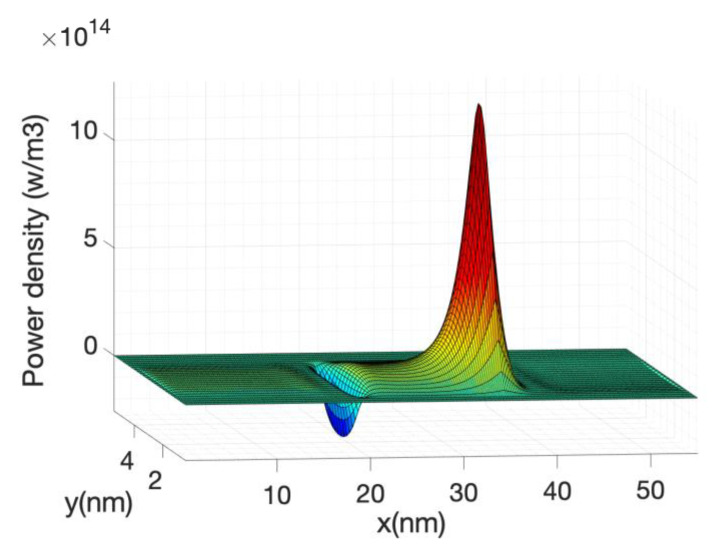
Power density profile used as heat source (Joule heating) in the heat equation. Vg = 0.7 V.

The heating of the lattice due to the energy transfer from the electron system into the phonon system produces an increase in the electron-phonon scattering rate and therefore has the effect of reducing the current. This reduction is substantial (at Vg = 0.7 V), compared with the case in which the phonon system is considered at a temperature of 300 K or no self-heating. This is clearly shown in the current voltage characteristics depicted in Figure 7. However, the effect is even large if we neglected hot electron effects and assumed that the electron system is in local equilibrium with the potential, i.e., assuming the Joule law. The Figure 7 shows a very large reduction in current when Joule heating is considered. The corresponding temperature profile and heat source profile for this case are shown in Figure 18 and Figure 19. The heat source in this case (Figure 20) is just proportional to the gradient of the electrostatic potential. Note that the maximum temperature (over 600 K) is substantially larger than the previous case, indicating that the Joule heat grossly overestimates the power dissipation inside small nanowire transistors.

Figure 19 shows the temperature along the device corresponding to the case in which the electron system dissipates the Joule power into the lattice but where we fixed the lattice temperature of 300 K in computing the scattering of electrons. In this case as there is not a reduction in the electron energy current the lattice temperature increases substantially, the maximum peaking around 1000 K. This shows the danger of considering only the energy transfer from the electrons to the lattice, but not the impact of the heated lattice on the electron system for small nano-transistors.

### 3.5. Perspectives and Challenges

In this section, we describe certain open issues and limitations of the simulation of carrier transport in nano-transistors relevant to our work. Even though substantial progress in nanowire transistor simulation has been achieved, reliable and unified models do not exist yet. The majority of simulation methodology has a simplified description of the contacts. Usually, the contacts have the same cross section as the active part of the device. This is very convenient when using the NEGF formalism as it requires a region of uniformity in order to properly inject and absorb carriers through self-energies. A more realistic description of the contacts would require [59] a broadening of the contacts relative to the device active region. This broadening, of course, would depend on the realistic geometry of the device and needs a large simulation domain as a large part of the extension regions would need to be added. Calculations using different source/drain geometries have been carried out in a ballistic context [95]. In the self-consistent NEGF/Poisson formalism used to simulate devices, the Fermi levels are fixed in the boundary of the source and drain and the potential is allowed to float to achieve charge neutrality. This fixing of the Fermi level would be more accurate as the electrical neutrality in the contact should not be affected by the device active region, as the contacts will ultimately represent reservoirs in equilibrium. However, these reservoirs could be made of or connect 1D, 2D and 3D electron states. In addition, the broadening of the contacts will create new challenges from a computational point of view as scattering needs to be considered in those contacts in order to inject electrons in the narrow regions with the proper equilibrium distribution, the proper quantum states and energies. The transition region between the simulated contacts and the narrow device region will induce unwanted reflections if the transition is abrupt, therefore a smooth change in cross section, from wide to narrow channel is required in conjunction with decoherence and randomization due to electron-phonon scattering. A proper smoothness of the transition will require the use of the finite element method [96] to treat the change of geometry in an intrinsic way. However, this will stretch the use of the recursive algorithm [97], which is the pinnacle of success in NEGF computational versatility. Electron-electron scattering in the interfaces and contacts could play a role but it would be challenging to introduce into reliable, computationally manageable and even theoretical models [51,52]. In addition, electrons in the channel experience remote interactions from interfaces and contacts such as soft optical interface phonon scattering [53,54] and remote Coulomb scattering. The latter references indicate that these mechanisms could have substantial impact on device performance. The modelling and description of these mechanisms are not unique and represent a challenge due to the reduced and changing dimensionality of the nanostructure. 

The present study has not considered the influence of atomistic effects on electro-thermal modelling, such as stray discrete impurities, trapped charge at the interfaces or discrete effects of the atomistic interfaces, although we have made extensive studies of these effects in purely electron transport modelling (e.g., [11,79]). However, the high surface to volume ratio for nanowires raises the issue of electronic interface states and their effect on electro-thermal properties which deserves future scrutiny. In addition, the role of localised phonon modes at the interfaces could lead to important thermal channels and local non-equilibrium phonon effects.

However, there is one aspect of simplification that needs caution: it has been usual in most studies to neglect the real part of the self-energies. Unfortunately, this is not justified in general. For example, the retarded self-energy has to satisfy causality requirements and these lead to Kramers-Kronig relations (Hilbert transform) that relate the real part of the self-energy to the imaginary part. As we have shown, elsewhere (see Appendix G and references herein), neglect of the real part may lead to serious violations of the spectral density sum rule and possible violations of charge conservation. Significant differences may be demonstrated in device modelling [77]. In general, the real part of the retarded self-energy leads to energy-dependent level shifts and may lead to bifurcation of the spectral density of states. The drawback is that the procedure for the full self-energy (within the self-consistent Born approximation) is very much more compute-intensive (further details in references Appendix G).

The use of a heat equation in this work and others as an alternative to more complex methodologies and moving towards more computational efficient methods is promising. The similarity in results with other methodologies demonstrated in ref [94] is encouraging in spite of the strong dissimilarities between methodologies. Their use of NEGF description for phonon transport and the full phonon dispersion law, instead of the simple heat equation, made the methodology extremely computationally demanding and therefore limiting its scope. We use a very simplified version of the phonon scattering mechanisms [85,98] that has been proven to produce similar values and trends for low field mobilities [76] and which has been widely used in the Monte Carlo/Greenwood-Kubo methodologies and adapted for nanowire [98]. The calculation of phonon modes in confined nanostructures is also a challenge, as they strongly depend on the boundary conditions used [99]. In our study, the acoustic deformation potential has been parameterised to partially take care of the decrease in mobility for narrow nanowire following [86,98]. Using a heat equation has the advantage of extending thermal simulations in larger regions surrounding the device and interconnects. In addition, interfaces and boundaries between materials of different thermal conductivities can be handled. This opens up the possibility of using more realistic thermal environments [100,101] and compact models as thermal resistance. Thermal conductivities which vary with the temperature could also be incorporated. Simple phonon balance equations can be used instead of the heat law if necessary, alternatively, different heat equations for different phonon types (for example separately for polar and acoustic) could also be used and this could partially address heat transport through interfaces and phonon-phonon interaction. Finally, thermal transport processes in low-dimensional and non-trivial spatial topology nanostructures (so-called “holey” materials) are becoming important [102]for applications in thermo-electrics, photonics and batteries . Reference [102] provides a detailed review of this challenging field and showed that thermal conductivity measurements at room temperature are considerably lower than the Casimir limit for structures smaller than a few hundreds of nanometers. The traditional continuum models of thermal diffusion and conduction no longer apply. The power of the spatially resolved NEGF methodology may prove important in that context.

## 4. Conclusions

In this paper, we revised and reviewed some of the quasi-classical and quantum methodologies used for the electrothermal simulation of nanoelectronics devices. Details and insights about the physics behind the formalism were also provided and these were complemented with the relevant literature and clarifying appendixes. The rest of this work focused on the application of a blended electrothermal methodology to a gate all around nanowire transistor. 

Specifically, we have carried out electrothermal simulations of a narrow gate all-around nanowire transistor using a quantum transport formalism. The non-equilibrium Green function formalism has been used to describe the electron transport and the electron-phonon scattering is described within the self-consistent Born approximation 

We have modelled the lattice heating by a Fourier law, and used as a heat source the true loss or gain of energy of the electrons (hot electrons) to the phonons.

These simulations have been compared with the use of the Joule law as a heat source. The drain on-current is reduced around 30% when the hot phonon source is used and 70%, compared to when the Joule law alone is used. 

We have found that at low gate bias a slightly cooling of the source region by a few degrees occurs. The effect is large at an intermediate gate bias that depends on the electron-phonon scattering strength and the length of the source. This effect might be used for cooling the device if the source material and the dimension of the device are properly engineered.

At high gate bias, the injected electrons go straight to the drain region without the need of phonon absorption as the source drain barrier height is negligible. The dissipation of energy from the high energy electrons injected from the source induces an overall heating in the active region of the device. This process is more pronounced in the drain side as has been confirmed by the simulations. This effect becomes larger as the current increases. However, as the lattice heats, the average phonon energy or the equivalent temperature of the phonon system increases, and as a consequence, the electron-phonon scattering rate increases, the electron current is decreased from its value when the lattice is at room temperature. This results in less heating of the lattice. Our simulation produces a maximum device temperature of 400 K at large gate bias.

We have also investigated the case that the electrons are in local equilibrium with the lattice and used the Joule power as a source of the heat equation, this approximation is only valid for large devices. This produces a large heating of the lattice and consequently a large decrease in the drain current. The maximum temperature is approximate 600 K. 

The methodology presented in this paper is computationally efficient and allows the accurate prediction of the effect of self-heating in small devices and produces very similar results to more sophisticated but computationally intensive methodologies. As indicated, some simple models can reproduce the trends of more sophisticated ones in a more computational efficient way; however, these models require verification or at least justification of the principle used. These models usually provide a simpler and concise explanation of the behaviour of the devices as they used few physical parameters. This work is a small step in this direction of the search for more simplified and phenomenologically-based models allowing the incorporation of more phenomena and larger parts of the device environment in the simulation domain. The solution of the 3D heat equation has a similar computational complexity to the 3D Poisson equations, which are solved quite efficiently by iterative methods. Therefore, quantum transport can be naturally connected with self-heating and thermal transport in a large domain. This provides more realistic boundary conditions, i.e., boundaries at room temperature should be far away from the active region of the device. For a device, designer/engineer accuracy and speed are equally important as substantial numbers of devices and conditions would need to be considered before an optimum decision is taken. 

It is anticipated that our methodology may find wide application in the design of power efficient nanodevices. 

## Appendix A. Local Density of States for Simple Systems

Here, we compare classical and simple quantum expressions for the LDOS of a simple model of the potential energy profile for a silicon NWFET of total length 55 nm; the channel is between 20 nm and 35 nm.

The gate bias is Vg = 0.2 V, with a drain bias of 0.6 V. We consider only a single sub-band for transport along the [100] direction. The quantum expression used is derived from (25) for a piece-wise constant potential as:

(A1) $$\rho_{LDOS}^{\;}\left(x,\varepsilon \right)=|\psi_{k\left(\varepsilon \right)}\left(x\right){|}^{2}\left(\frac{2m}{\pi^{\;}\hbar^{2}}\right)/\sqrt{\frac{2m\left(\varepsilon -V\left(x\right)-\varepsilon_{c}\right)}{\hbar^{2}}}\;$$

In the classical case, the term $|\psi_{k\left(\varepsilon \right)}\left(x\right){|}^{2}$ becomes unity. The inverse square root gives rise to very high values for the density of states at the sub-band edge. The strong fringing modulation in the quantum case derives from the strong reflections of electrons at source side and the drain side of the gate barrier.

For realistic semiconductor models, the orientation of the 1D axis is crucial (usually along the [100] direction) and multiple valleys must be considered. This leads to a superposition of 1D LDOS terms associated with different valleys. The full range of Δvalleys considered is shown in the iso-energy surfaces in k-space in Figure A2, showing the principal valleys for transport in the x-direction in orange and the other valleys in silver.

## Appendix B. Use of Self-Energies to Project Coupling of Contacts into Finite Device Domain

Consider the Green function operator $G_{total}\left(\varepsilon \right)$ for the open system comprising a finite nano-device connected through essentially open source (S) and drain contacts (D), which have well-defined temperatures and chemical potentials at the interface to the device (W). $G_{total}\left(\varepsilon \right)\;$satisfies the eigenvalue equation (I is a unit matrix):

(A2) $$(\varepsilon I-H_{total}\;)G_{total}{}_{\;}\left(\varepsilon \right)=I$$

In any representation (Equation (A3)), is a huge intractable matrix equation. In matrix operator form we may formally partition this Hamiltonian into a matrix of sub-Hamiltonians following reference [103], representing the finite device in interaction with the open source/drain contact domains. Consider the Green function *G_WW_* for the finite device (W) where electrons are injected or extracted at the surface of the Drain (D) and Source (S) contacts. The total Green function operator is partitioned into the matrix

(A3) $$G_{total}=\left[\begin{array}{ccc} G_{SS} & G_{SW} & G_{SD}\\ G_{SW} & G_{WW} & G_{WD}\\ G_{DS} & G_{DW} & G_{DD} \end{array}\right]$$

and we similarly partition the total Hamiltonian

(A4) $$H_{total}=\left[\begin{array}{ccc} H_{SS} & H_{SW} & 0\\ H_{SW}^{†}{}_{\;} & H_{WW} & H_{DW}^{†}\\ 0 & H_{DW} & H_{DD} \end{array}\right]$$

The source/drain contact regimes are represented by Green functions *G_SS_/G_DD_* and Hamiltonians *H_SS_/H_DD_,* The finite nanowire device (simulation region) is modelled by a Hamiltonian *H*_ww_, The transfer Hamiltonians are non-hermitian forms, *H_SW_,H_DW_*, which inject or extract carriers at the source contact:source extension boundary surface and at the drain contact: drain extension boundary surface. Introducing a matrix of the sub-domain Green functions (in an obvious notations) Equation (A3) becomes the matrix equation:

(A5) $$\left[\begin{array}{ccc} \varepsilon -H_{SS} & -H_{SW} & 0\\ -H_{SW}^{†}{}_{\;} & \varepsilon -H_{WW} & -H_{DW}^{†}\\ 0 & -H_{DW} & \varepsilon -H_{DD} \end{array}\right]\left[\begin{array}{ccc} G_{SS} & G_{SW} & G_{SD}\\ G_{SW} & G_{WW} & G_{WD}\\ G_{DS} & G_{DW} & G_{DD} \end{array}\right]=\left[\begin{array}{ccc} I & 0 & 0\\ 0 & I & 0\\ 0 & 0 & I \end{array}\right]$$

Solving the equations corresponding to the second column of the identity matrix in Equation (A5), it is simple to derive the Green functions for source and drain, *G_SW_ and G_DW_*:

(A6) $$G_{SW}=g_{SS}H_{SW}G_{WW}$$

(A7) $$G_{DW}=g_{DD}H_{DW}G_{WW}$$

(A8) $$G_{WW}={\left(\varepsilon -H_{WW}-\Sigma_{SW}-\Sigma_{DW}\right)}^{-1}$$

where

(A9) $$g_{SS}={\left(\varepsilon -H_{SS}\;\right)}^{-1};\;g_{DD}={\left(\varepsilon -H_{DD}\;\right)}^{-1}$$

and the contact-coupling self-energies are:

(A10) $$\Sigma_{SW}=H_{SW}^{†}g_{SS}H_{SW};\;\Sigma_{DW}=H_{DW}^{†}g_{DD}H_{DW}\;$$

Equation (A8) illustrates how to project out a finite domain Green function from the infinite open system by introducing contact self-energies

(A11) $$\Sigma_{contacts}=\Sigma_{SW}+\Sigma_{DW}$$

at the boundaries. The retarded and advanced Green functions are retrieved by adding ± iη (η>0) to the energy ε. A similar scheme applies to the lesser and greater Green functions. The details depend on the system.

## Appendix C. The Keldysh NEGF Equations

The Keldysh formalism describes a number of different NEGF, only 3 of which are independent. Here, it is assumed that three independent Green functions are $G^{R}$, $G^{A},\;G^{<}$. In matrix operator form, the steady-state Green functions satisfy:

(A12) $$G^{R}={\left(\varepsilon +i\eta -H_{0}-H_{ext}-\Sigma^{R}{}_{\;}\right)}^{-1}=G_{0}^{R}{}^{\;}+G_{0}^{R}\Sigma^{R}G^{R}$$

(A13) $$G_{0}^{R^{-1}}={\left(\varepsilon +i\eta -H_{0}-H_{ext}\right)}^{\;}$$

(A14) $$G^{A}=G^{R^{†}}$$

(A15) $$G_{}^{<}{}^{\;}=G_{\;}^{r}\Sigma^{<}G^{A}$$

(A16) $$G_{}^{>}{}^{\;}=G_{\;}^{r}\Sigma^{>}G^{A}$$

Here, for convenience, we include any confinement potential energy *H*_c_(r) into the term *H*_ext,_

(A17) $$H_{ext}\to H_{c}\left(r\right)+e\phi \left(r\right)$$

which makes explicit the applied electrostatic potential.

Equation (A12) is the first Keldysh equation for the retarded Green function *G*^R^; it describes the dynamical states of the system (current carrying states, applied field corrections, confinement potential renormalisation, bound states, resonances and lifetime broadening of states). Equation (A15) is the second Keldysh equation describing the statistical distribution of states due to scattering (analogous to the Boltzmann equation). H*_ext_*(r) is the total potential energy comprising, confinement potential including possible interface roughness, plus the electrostatic potential energy eϕ(r) determined self-consistently from Poisson’s equation.

A connection to the Boltzmann transport equation may be made by defining the relative and centre coordinates *r*_rel_ and *R*, respectively:

(A18) $$r_{rel}=r-\;r\;{}^{\prime };\;R=\left(r+\;r\;{}^{\prime }\right)/2$$

Assuming the Hamiltonian for the electron kinetic energy is a function of the momentum operator p, and by applying the inverse Fourier transform of the relative space variable into momentum space, after some manipulation, Equations (A12)–(A19) may be used to give:

(A19) $$\{v\left(p\right)\cdot \frac{\partial }{\partial r}-\frac{\partial }{\partial r}\left\{H_{c}\left(r\right)+e\phi \left(r\right)\right\}\cdot \frac{\partial }{\partial p}\}G^{<}\left(p,R,\varepsilon \right)\;={\;\Sigma }^{>}\left(p,R,\varepsilon \right)G^{<}\left(p,R,\varepsilon \right)-{\;\Sigma }^{<}\left(p,R,\varepsilon \right)G^{>}\left(p,R,\varepsilon \right)$$

Equation (A19) has close similarity to the Boltzmann Equations (4) and (6), but $G_{}^{<}$ and $G_{}^{>}$ are generalised Wigner distributions similar to *f*(p,R) and *(1-f(*p,r*))* in semi-classical theory. The scattering integral on the RHS of Equation (A19) acquires the Boltzmann like exclusion principle structure *f(1-f)* when we deconstruct the self-energies $\Sigma_{}^{<}\propto G_{}^{<},\;\Sigma_{}^{>}\propto G_{}^{>}$ as shown in Appendix D.

Equation (A19) is difficult to solve compared to the standard NEGF spatial matrix elementenergy formulation. Discretising the space dimensions and taking the simple difference form of *H*_0_ yields a set of coupled matrix equations that may be solved recursively. For nanowire devices, the problem is sufficiently tractable to be solved quickly on a laptop. 2D or full 3D is much more computationally expensive.

## Appendix D. Phonon Green Functions and Electron-Phonon Self-Energies

To fully specify the basic Equations (A12)–(A16), we must construct the self-energies for the contacts $\Sigma_{contacts}^{<},$ $\Sigma_{contacts}^{R}\;$and construct the self-energies for electron-phonon scattering using the phonon Green functions *D*^R^ and *D*^<^. In the conventional decoupling approximation, we may assign the phonon Green functions in their local equilibrium forms. The decomposition equations are in *symbolic* operator form [74]:

(A20) $$\Sigma^{R}=G^{R}D^{R}+G^{R}D^{<}+G^{<}D^{R}$$

(A21) $$\Sigma^{<}=G^{<}D^{<}$$

where the matrix products involve an implicit energy convolution integral of the form

(A22) $$\Sigma \left(\varepsilon \right)\sim \int d{\varepsilon^{\prime }}^{\;}G\left(\varepsilon -\;\varepsilon \;{}^{\prime }\right)D\left(\varepsilon^{\prime }\right)$$

Several authors have neglected the third term on the RHS of Equation (A20), which can be fatal as it controls the Pauli exclusion, as pointed out in detail by Kubis and Vogel [104,105].

Here, we give explicit expressions for the lowest order self-consistent expression for the electron-phonon “lesser” self-energy written as a sum over the phonon branches *b* with energy $\hbar \omega_{bq}$, where q denotes the phonon wave vector.

It is assumed, for generality, that the phonon distributions are at individual lattice temperatures *T_b_*. A 3D description is given for generality.

The free phonon Hamiltonian is:

(A23) $$H_{ph}=\underset{b}{\sum }\;\int d^{3}q\left(N_{bq}+\frac{1}{2}\right)\;\hbar \omega_{bq}$$

where $N_{bq}=a_{bq}{}^{†}a_{bq}$ is the phonon number operator in terms of the usual Bosonic annihilation and creation operators $a_{bq},\;a_{bq}{}^{†}$, respectively. The condition that the phonons comprise a thermal equilibrium heat bath (possibly locally) requires us to define the phonon Green functions in terms of the following transformed operators (see for example [71])

(A24) $$A_{bq}\equiv (a_{bq}+a_{b-q}{}^{†});\;A_{bq}^{†}\equiv (a_{b-q}+a_{bq}{}^{†})$$

in the Heisenberg picture

(A25) $$A_{bq}\left(t\right)=A_{b-q}^{†}\left(t\right)=\exp[-i\omega_{bq}t]a_{bq}+\exp[i\omega_{bq}t]a_{b-q}^{†}{{}_{\;}}_{\;}{}^{\;}$$

The basic double-time phonon Green function is time-ordered on the Keldysh contour (using the Boson time-ordering rules)

(A26) $$D_{b}=<T_{Keldysh}A_{bq}\left(t\right)A_{b-q}^{†}\left(t\right){>}_{0eq}$$

The relevant piece-wise time-ordered phonon Green functions are

(A27) $$D_{b}^{<}\left(q,\varepsilon \right)=D_{b}^{>}\left(-q,\varepsilon \right)=-2\pi i\left\{n_{bq}\delta \left(\varepsilon -\hbar \omega_{bq}\right)+\left(n_{bq}+1\right)\delta \left(\varepsilon +\hbar \omega_{bq}\right)\right\}$$

(A28) $$D^{R}\left(q,\varepsilon \right)=\{{\left(\varepsilon +i\eta -\hbar \omega_{bq}\right)}^{-1}-{\left(\varepsilon +i\eta +\hbar \omega_{bq}\right)}^{-1}\;$$

The basic electron-phonon interaction [71] is

(A29) $$H_{int}\left(r,t\right)=\psi^{†}\left(r,t\right)\underset{b}{\sum }\;\int \frac{d^{3}q}{{\left(2\pi \right)}^{3}}U_{b}\left(q\right)e^{iq.r}\{a_{bq\left(t\right)}+a_{b-q}{}^{†}\left(t\right)\}\psi^{\;}\left(r,t\right)$$

U_b_(q) is the matrix element of the coupling potential for the *b*th mode phonon in the lattice wave-vector representation. In many-body perturbation theory the lowest self-consistent approximation for the total electron-phonon self; energies are obtained in the self-consistent Born approximation (SCBA), using Equations (A21) and (A27):

(A30) $$\begin{array}{cc} \Sigma^{<}=G^{<}D^{<}\to \Sigma^{<}( & \left.r_{1},r_{2},\varepsilon \right)\\ = & \sum_{b}\int \frac{d^{3}q}{{\left(2\pi \right)}^{3}}{\left|U_{b}\left(q\right)\right|}^{2}e^{iq\cdot \left(r_{1}-r_{2}\right)}\int \frac{d\varepsilon^{\prime }}{2\pi }D^{<}\left(q,\varepsilon^{\prime }\right)G^{<}\left(r_{1},r_{2},\varepsilon -\varepsilon^{\prime }\right)\\ = & \sum_{b}\int \frac{d^{3}q}{{\left(2\pi \right)}^{3}}{\left|U_{b}\left(q\right)\right|}^{2}e^{iq\cdot \left(r_{1}-r_{2}\right)}\times \left\{n_{bq}G^{<}\left(r_{1},r_{2},\varepsilon -\hbar \omega_{bq}\right)+\right.\\ \; & \left(n_{bq}+1)G^{<}\left(r_{1},r_{2},\varepsilon +\hbar \omega_{bq}\right)\right\}\alpha \end{array}$$

This may be compared with the semi-classical Equation (7). Here, the phonon distributions are assumed to be Bose-Einstein at temperature *T*_b_:

(A31) $$n_{bq}={\left(\exp\left[\frac{\hbar \omega_{bq}}{k_{B}T_{b}}\right]-1\right)}^{-1}$$

Here, the lattice temperature is potentially allowed to be different from the ambient temperature due to heat dissipation in the non-equilibrium device.

The SCBA also yields $\Sigma^{R}$ which assumes a much more complicated form, again carrying out the implicit convolution integral over energy, using Equatons (A20) and (A28):

(A32) $$\Sigma^{R}=G^{R}D^{R}+G^{R}D^{<}+G^{<}D^{R}\to \Sigma^{R}\left(r_{1},r_{2},\varepsilon \right)\;=\underset{b}{\sum }\;\int \frac{d^{3}q}{{\left(2\pi \right)}^{3}}|U_{b}\left(q\right){|}^{2}\;e^{iq{.(r}_{1}-r_{2})}\times \{n_{bq}G^{R}\left(r_{1},r_{2,}\varepsilon +\hbar \omega_{bq}\right)\;+(n_{bq}+1)G^{R}\left({\text{r}}_{1},{\text{r}}_{2,}\varepsilon -\hbar \omega_{b\text{q}}\right)\;\;\;\;+\left(\frac{1}{2}\right)(G^{<}\left({\text{r}}_{1},{\text{r}}_{2,}\varepsilon -\hbar \omega_{b\text{q}}\right)-G^{<}\left({\text{r}}_{1},{\text{r}}_{2,}\varepsilon +\hbar \omega_{b\text{q}}\right)\;-\left(\frac{i}{2\pi }\right)\int^{\text{​}}\text{d}\varepsilon^{\prime }\left\{P\frac{G^{<}\left({\text{r}}_{1},{\text{r}}_{2,}\varepsilon -\varepsilon^{\prime }\right)}{\varepsilon^{\prime }+\hbar \omega_{b\text{q}}}-P\frac{G^{<}\left({\text{r}}_{1},{\text{r}}_{2,}\varepsilon -\varepsilon^{\prime }\right)}{\varepsilon^{\prime }-\hbar \omega_{b\text{q}}}\right\}\}$$

Note the coupling to G^<^ and principal-part (*P*), integral over the intermediate energy, that derives from Equation (A28) for *D^R^*. The above equations use the identity

(A33) $$\underset{\eta \to 0^{+}}{\lim}\frac{1}{\varepsilon -\varepsilon^{\prime }+i\eta }=\frac{P}{\varepsilon -\varepsilon^{\prime }}-i\pi \delta \left(\varepsilon -\varepsilon^{\prime }\right)$$

The principal-part integrals cancel out for *elastic* scattering: $\omega_{bq}\to 0$. It should be noted that if the interaction potential is independent of q and if the phonon energies are dispersionless, then the self-energy is diagonal.

It is tempting to replace the Green functions *G^<^* in Equation (A22) by the unperturbed forms $G_{0}^{<}$, which gives essentially the semi-classical Equation (7); however, the standard Born approximation fails the continuity equation which for the steady state assumes the form:

(A34) $$\nabla \cdot J\left(r\right)=\int d\varepsilon \left(\frac{e}{\hbar }\right)<r\left|\left\{G^{R}\Sigma^{<}-\Sigma^{<}G^{A}+G^{<}\Sigma^{A}-\Sigma^{R}G^{<}\right\}\right|r>=\int d\varepsilon <r\left|I_{current}\right|r>$$

where we recognise the current operator *I_current_* [57]

(A35) $$I_{current}\left(\varepsilon \right)=\left(\frac{e}{\hbar }\right)\left\{G^{R}\Sigma^{<}-\Sigma^{<}G^{A}+G^{<}\Sigma^{A}-\Sigma^{R}G^{<}\right\}$$

## Appendix E. Elastic Phonon Scattering Gives no Heat Dissipation

Using the specific forms for the self-energies (Equations (A30) and (A32)) for the elastic scattering limit of acoustic phonon scattering (high temperature model), we may demonstrate that Equation (A30) vanishes within the device volume. The coupling interaction is:

(A36) $$|U_{\;}\left(q\right){|}^{2}=\frac{\hbar \Xi_{d}^{2}}{2\rho_{d}\omega_{q}}q^{2}$$

where the acoustic phonon dispersion relation is assumed to be the linear form

(A37) $$\omega_{q}=v_{s}q$$

where v*_s_* is the speed of sound in the channel. At high temperatures, the Bose-Einstein functions are given in the equipartition limit as

(A38) $$n_{q}\sim \left(n_{q}+1\right)\sim \frac{k_{B}T}{\hbar \omega_{q}}$$

Using Equation (A38) and the dispersion relation (A37) the lesser self-energy becomes diagonal:

(A39) $$\Sigma^{<}\left(r_{1},r_{2},\varepsilon \right)=\int \frac{d^{3}q}{{\left(2\pi \right)}^{3}}|C{|}^{\;}\;e^{iq{.(r}_{1}-r_{2})}G^{<}\left(r_{1},r_{2,}\varepsilon \right)=\left|C\right|G^{<}\left(r_{1},r_{1,}\varepsilon \right)\delta \left(r_{1}-r_{2}\right)$$

(A40) $$\left|C\right|=\frac{\Xi_{d}^{2}k_{B}T}{\rho_{d}v_{s}^{2}}$$

In a similar fashion, using Equation (A22), it is found that $\Sigma^{R}$ decouples from $G^{<}$ to give a diagonal form:

(A41) $$\Sigma^{R}\left(r_{1},r_{2},\varepsilon \right)=\left|C\right|G^{<}\left(r_{1},r_{1,}\varepsilon \right)\delta \left(r_{1}-r_{2}\right)$$

Inserting the diagonal Equations (A39) and (A46) into Equation (A34) gives

(A42) $$\nabla \cdot J\left(r\right)=\int d\varepsilon \left(\frac{e}{\hbar }\right)<r|(G^{R}-G^{A})\Sigma^{<}+\left(\Sigma^{A}-\Sigma^{R}\right)G^{<}|r>\;=\int d\varepsilon \left(\frac{e}{\hbar }\right)<r|(G^{R}-G^{A})\left|C\right|G^{<}+\left|C\right|\left(G^{A}-G^{R}\right)G^{<}|r>=0$$

Similarly, the divergence of the total energy current density (Equation (67)) may be shown to vanish for elastic acoustic phonon scattering:

(A43) $$\nabla \cdot J_{\mathrm{Energy}}\left(r\right)=\int d\varepsilon \;\varepsilon <r|(G^{R}-G^{A})\Sigma^{<}+\left(\Sigma^{A}-\Sigma^{R}\right)G^{<}|r>\;=\int d\varepsilon \;\varepsilon <r|(G^{R}-G^{A})\left|C\right|G^{<}+\left|C\right|\left(G^{A}-G^{R}\right)G^{<}|r>=0$$

## Appendix F. Detailed Balance

Integrating Equation (A34) over all space in the device we obtain

(A44) $$\int d^{3}r^{\;}\;\nabla \cdot J\left(r\right)=\int d\varepsilon \;\mathrm{Tr}\left[I_{current}\left(\varepsilon \right)\right]=0$$

The expression

(A45) $$\mathrm{Tr}\left[I_{current}\left(\varepsilon \right)\right]=\mathrm{Tr}\left[G^{<}\Sigma^{>}-G^{>}\Sigma^{<}\right]$$

is basically a trace over the RHS of Equation (A19) and is similar to the principle of detailed balance in semi-classical theory, but here the trace does not vanish in general to satisfy Equation (A20). It is the energy integral over Equation (A45) that vanishes. We note there is a wealth of physics hidden in Equation (A45), especially if the contact self-energies are made explicit.

For inelastic scattering, the power dissipation to the lattice is

(A46) $$P\left(r\right)=\int d\varepsilon \;\varepsilon \sum_{r^{\prime }}\left\{G^{<}\left(r,r^{\prime }\varepsilon \right)\Sigma^{>}\left(r,r^{\prime },\varepsilon \right)-G^{>}\left(r,r^{\prime }\varepsilon \right)\Sigma^{<}\left(r,r^{\prime },\varepsilon \right)\right\}$$

## Appendix G. Kramers-Kronig Relations

There have been many instances of device modelling studies that have neglected the real part of the retarded self-energy often on the grounds that is a “small” correction. In fact this cannot be the case because the self-energy has a definite analyticity structure arising from causality and this leads to a Kramers-Kramers-Kronig relation (Hilbert transform) between the real part Δ and the imaginary part Γ of the self-energy:

(A47) $$\Delta \left(\varepsilon \right)=-\frac{P}{\pi }\int_{-\infty }^{\infty }d\varepsilon \prime \frac{\Gamma \left(\varepsilon^{\prime }\right)}{\varepsilon^{\prime }-\varepsilon }$$

As can be seen from Equation (47), the calculation of the real part requires an extra energy integration loop for each energy resulting in an additional computational load. Our studies have shown that this leads to significant differences in device modelling predictions from those neglecting the real part. In particular, the local density of states may be seriously mis-calculated and the spectral density sum rule [71] is violated. There are then significant errors caused in electro-thermal predictions. Detailed discussion may be found in the references [77,106,107,108]. In addition, it has been demonstrated that the neglect of the real part of the self-energy, for simulation of low dimensional nanostructures induces a violation of charge conservation of 5% [108].
